# Investigating the Impact of Irrigation on Malaria Vector Larval Habitats and Transmission Using a Hydrology‐Based Model

**DOI:** 10.1029/2023GH000868

**Published:** 2023-12-10

**Authors:** Ai‐Ling Jiang, Ming‐Chieh Lee, Prashanth Selvaraj, Teshome Degefa, Hallelujah Getachew, Hailu Merga, Delenasaw Yewhalaw, Guiyun Yan, Kuolin Hsu

**Affiliations:** ^1^ Department of Civil and Environmental Engineering Center for Hydrometeorology and Remote Sensing University of California Irvine Irvine CA USA; ^2^ Department of Population Health and Disease Prevention School of Public Health Susan and Henry Samueli College of Health Sciences University of California Irvine Irvine CA USA; ^3^ Institute for Disease Modeling Bill and Melinda Gates Foundation Seattle WA USA; ^4^ School of Medical Laboratory Sciences Institute of Health Jimma University Jimma Ethiopia; ^5^ Tropical and Infectious Diseases Research Center (TIDRC) Jimma University Jimma Ethiopia; ^6^ Department of Medical Laboratory Technology Arbaminch College of Health Sciences Arba Minch Ethiopia; ^7^ Department of Epidemiology Institute of Health Jimma University Jimma Ethiopia

**Keywords:** hydrologic modeling, malaria transmission modeling, irrigation, surface water ponding, vector borne disease, vector larval habitat

## Abstract

A combination of accelerated population growth and severe droughts has created pressure on food security and driven the development of irrigation schemes across sub‐Saharan Africa. Irrigation has been associated with increased malaria risk, but risk prediction remains difficult due to the heterogeneity of irrigation and the environment. While investigating transmission dynamics is helpful, malaria models cannot be applied directly in irrigated regions as they typically rely only on rainfall as a source of water to quantify larval habitats. By coupling a hydrologic model with an agent‐based malaria model for a sugarcane plantation site in Arjo, Ethiopia, we demonstrated how incorporating hydrologic processes to estimate larval habitats can affect malaria transmission. Using the coupled model, we then examined the impact of an existing irrigation scheme on malaria transmission dynamics. The inclusion of hydrologic processes increased the variability of larval habitat area by around two‐fold and resulted in reduction in malaria transmission by 60%. In addition, irrigation increased all habitat types in the dry season by up to 7.4 times. It converted temporary and semi‐permanent habitats to permanent habitats during the rainy season, which grew by about 24%. Consequently, malaria transmission was sustained all‐year round and intensified during the main transmission season, with the peak shifted forward by around 1 month. Lastly, we evaluated the spatiotemporal distribution of adult vectors under the effect of irrigation by resolving habitat heterogeneity. These findings could help larval source management by identifying transmission hotspots and prioritizing resources for malaria elimination planning.

## Key Points

- An agent‐based malaria model was coupled with a hydrologic model to spatially simulate transmission by resolving habitat heterogeneity
- The coupling framework enhanced larval habitat area variability which resulted in a lower malaria transmission prediction
- Irrigation sustained transmission year‐round, intensifying and shifting the peak forward by 1 month from the original period

## 1 Introduction

Malaria is a deadly disease caused by parasites transmitted to humans through the bites of infected female *Anopheles* mosquitoes. It is particularly acute in sub‐Saharan Africa and remains one of the region's most pressing public health challenges. About 95% of malaria cases and 96% of deaths in 2021 were recorded in sub‐Saharan Africa (World Health Organization, 2022). A combination of accelerated population growth and arid conditions worsened by climate change has inevitably created pressure on food security (Ward et al., 2016). This drives the development of several regional irrigation schemes, which have been associated with increased malaria risk (Mangani et al., 2022). In past studies, the association has been chiefly demonstrated by statistical relationships based on field observations (Haileselassie et al., 2021; Kibret et al., 2014; Ondeto et al., 2022). However, these relationships may only be represented in some environmental settings because field observations are made at a limited number of discrete points in space and time.

Malaria modeling has the potential to complement field studies by exploring hypothetical scenarios and making a priori predictions that can inform intervention strategies. Originating from the basic Ross‐Macdonald model (Ross, 1908), many compartmental models have inherited its simplifying assumptions, such as homogeneous biting and well‐mixing of hosts and vectors, which is a shortcoming of representing the vectors and hosts as a population group rather than individuals (Reiner et al., 2013). In cases where spatial heterogeneity and stochasticity of disease progression are essential such as in a low‐transmission setting, agent‐based models (ABMs) can provide an explicit representation of individual actions and responses (N. R. Smith et al., 2018). Examples of advanced ABMs include Epidemiological MODeling (EMOD) (Eckhoff, 2011), OpenMalaria (T. Smith et al., 2006), and a model developed at Imperial College (Griffin et al., 2010). While widely used in malaria intervention studies (Galactionova et al., 2021), these models tend to neglect the larval habitat representation (Griffin et al., 2010; T. Smith et al., 2006) or rely only on rainfall to quantify larval habitats (Eckhoff, 2011).

A recent study by Smith et al. demonstrated that using a hydrologic model to simulate habitat availability can uncover more complex patterns in climatic suitability for malaria transmission than applying a rainfall threshold (M. W. Smith et al., 2020). This is because the formation of larval habitats is heavily influenced by hydrologic processes, which are highly non‐linear and spatially variable. In a hydrologic cycle, rainfall is partitioned into infiltration and surface runoff based on the soil type. Depending on the topography and surrounding vegetation, the resulting surface runoff will accumulate or drain. The persistence of the ponded water can also be influenced by evapotranspiration which varies with land use type. Besides rainfall, breeding sites can develop from groundwater, irrigation, reservoirs, and around dams. In irrigated settings, irrigation varies seasonally with crop production. Within a season, irrigation changes with local soil saturation and crop water use. The spatiotemporal variability of irrigation can result in habitats with different persistence and productivity. This diversity in habitat characteristics engenders the breeding of different species and complicates the pattern of adult mosquito density and malaria transmission intensity (Frake et al., 2020; Hardy et al., 2013; Munga et al., 2006). Therefore, incorporating hydrologic processes into malaria modeling to capture habitat heterogeneity is essential and can help provide better insights into how irrigation affects malaria transmission.

There have been attempts to represent surface hydrology in malaria transmission modeling with varying levels of complexity and success (Asare, Tompkins, & Bomblies, 2016). Most resort to a simple conceptual water balance model to determine the availability of water for larval habitats (Asare, Tompkins, Amekudzi, & Ermert, 2016; Montosi et al., 2012; Parham et al., 2012; Patz et al., 1998), while only a few have proposed more sophisticated hydrologic models that further consider canopy processes and subsurface flows (Bomblies et al., 2008; Le et al., 2018). Despite representing larval habitats more realistically, their malaria transmission component is often less comprehensive than advanced ABMs. Furthermore, none of these studies have investigated the impact of irrigation on malaria transmission.

In this study, we integrate a physical‐based hydrologic model, ParFlow‐Community Land Model (ParFlow‐CLM) (Ashby & Falgout, 1996; Jones & Woodward, 2001; Kollet & Maxwell, 2006; Maxwell, 2013; Maxwell & Kollet, 2008), with EMOD for a test site in Ethiopia. We chose EMOD because it is open‐source and can be easily modified to assimilate inputs from an external hydrologic model. We aim to demonstrate how incorporating hydrologic processes to estimate larval habitats can affect malaria transmission intensity and seasonality. Using the coupled model, we then examine the impact of an existing irrigation scheme on malaria transmission.

## 2 Materials and Methods

### 2.1 Study Site

**Figure 1:**
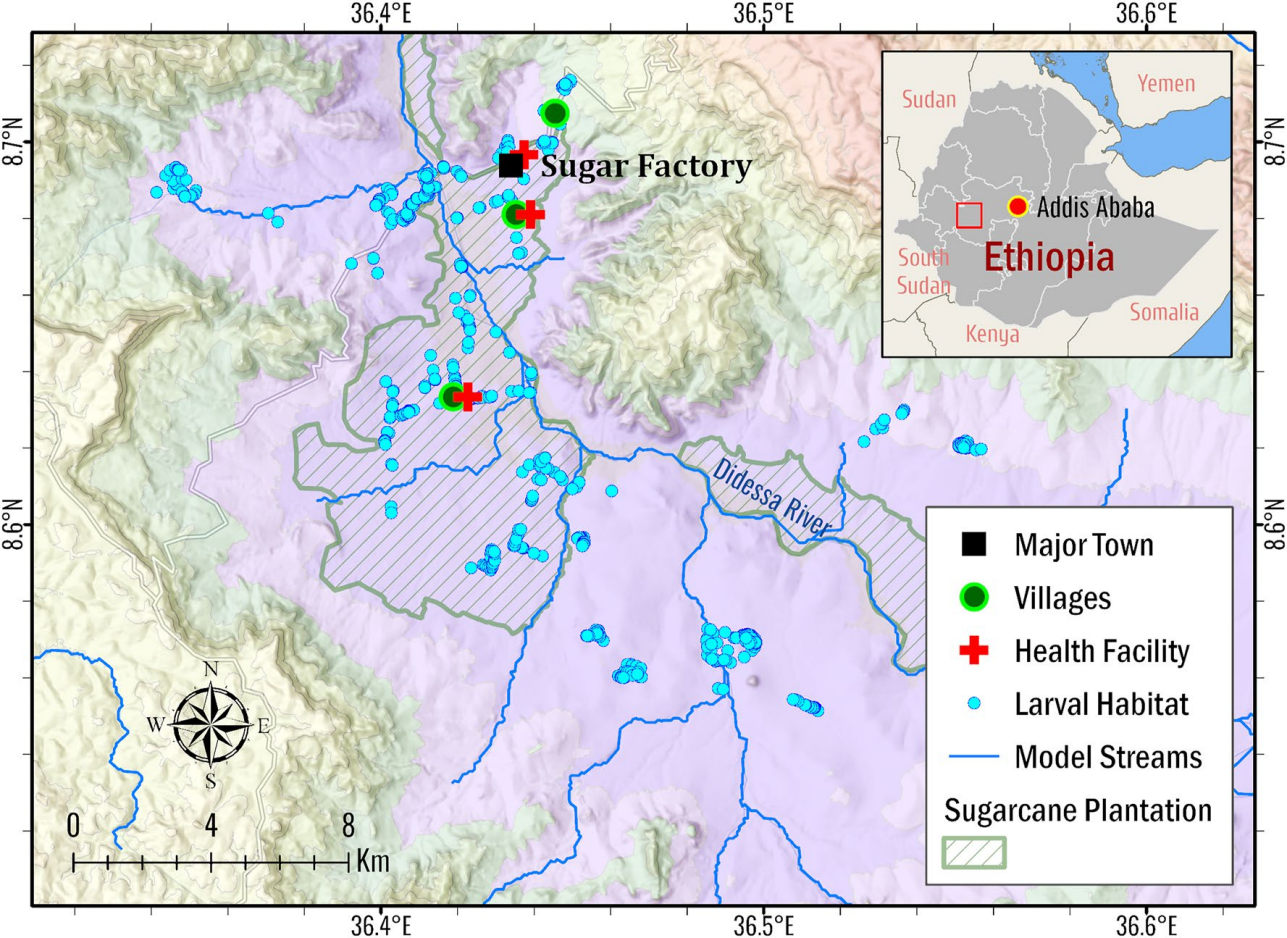
Arjo‐Didessa study site. The sugarcane plantation is demarcated in green within the study site. The surveyed larval habitats, represented by the blue markers, were used to estimate larval density and calibrate the hydrologic model. The three red crosses represent health facilities frequented by plantation workers from nearby villages and provide clinical data for Epidemiological MODeling calibration.

We conducted this study in the Arjo‐Didessa sugarcane plantation and its vicinity in the Didessa river valley, near Arjo town in Oromia Regional State, western Ethiopia (Figure 1). The study site includes a commercial sugar factory and an active irrigation area that relies on seasonal migrant workers for planting and harvesting. The site elevation ranges from 1,275 to 2,105 m above sea level, with a mean annual rainfall of 1,560 mm from 1994 to 2020 (Figure S1 in Supporting Information S1). The primary rainy season is between May and October, and the dry season occurs for the rest of the year. Monthly average relative humidity varies widely from around 40%–80%. It follows the rainfall pattern, while the monthly average temperature ranges from 19 to 24°C and is lower in the rainy season than in the dry season (Figure S2 in Supporting Information S1). Flow and sprinkler irrigation are commonly practiced in the plantation (Fikadu, 2015). The irrigation water is sourced from Didessa River. Due to poor drainage caused by the low permeability of the extensive heavy clay, the area is a perennial hotspot for larval habitats and is known to be malarious (Demissew et al., 2020; Hawaria et al., 2019). Malaria prevalence in this area is less than 3%, and transmission is seasonal, with cases peaking between September to December (Hawaria et al., 2019). *Anopheles arabiensis* is the primary malaria vector in this area. Local clinical malaria data shows that both major malaria parasites in Ethiopia, *Plasmodium falciparum* and *Plasmodium vivax*, co‐exist with equal incidences but significant seasonality (Hawaria et al., 2019).

### 2.2 Data Collection

ParFlow‐CLM requires climate data, including rainfall, short‐wave radiation, long‐wave radiation, air temperature, surface pressure, specific humidity, and wind speeds, to drive the hydrologic processes. The data were obtained from Precipitation Estimation from Remotely Sensed Information using Artificial Neural Networks‐Cloud Classification System‐Climate Data Record (PERSIANN‐CCS‐CDR) (Sadeghi et al., 2021) and the Fifth Generation European Center for Medium‐Range Weather Forecasts Reanalysis (ERA5) (Hersbach et al., 2023). Air temperature, humidity, and long‐wave radiation were adjusted to account for the mismatch in surface elevation between ERA5 and the study area, following the method by Cosgrove et al. (2003). For processes at the land surface, topography was obtained from a commercial global digital elevation model, ALOS World 3D (Takaku & Tadono, 2017) with additional hydro‐conditioning (Jiang, Hsu, et al., 2023), and land cover information was extracted from Global Land Cover Mapping Project (GlobeLand30), which is based on Landsat and Chinese HJ‐1 satellite images (Chen et al., 2015). To characterize the subsurface, the top 2 m from the surface was assumed to be soil comprising four layers and soil properties were referenced from the SoilGrids250m TAXOUSDA data set (Hengl et al., 2017). Parameters for the permeable subsoil layer above the bedrock (e.g., regolith and sediment deposits) were defined using GLHYMPS 2.0 (Gleeson et al., 2014). The bottom of the subsoil layer was delineated based on the depth to bedrock data from SoilGrids250m (Hengl et al., 2017). The climate inputs for EMOD include rainfall, air temperature, land temperature, and humidity. The rainfall data were similarly obtained from PERSIANN‐CCS‐CDR, and the others were extracted from ERA5. The list of model input data can be found in Table S1 in Supporting Information S1.

Besides publicly available data, several field surveys were conducted to support model development (Table S2 in Supporting Information S1). In previous studies, larval habitat data were collected over seasonal campaigns from 2017 to 2021, with a total of 769 sampled points (Hawaria et al., 2020). The larval habitat survey was conducted within 500 m of the village boundaries and sugarcane plantations. The surveyed larval habitat locations are shown in Figure 1. Mosquito larvae were sampled following the World Health Organization (WHO) standard larval survey procedure using a standard dipper (350 mL). Larvae were identified morphologically and sorted by genus as *Anopheles* or *Culex* in the field. From the survey, habitat locations within the hydrologic modeling domain were used to validate the predicted aquatic habitats simulated in ParFlow‐CLM, and larval density was used to determine the larval carrying capacity of the habitats in EMOD. In addition to the larval habitat survey, a land cover field survey was conducted in July 2021 in the study area. The survey was performed every 400 m along the perpendicular transects, spaced every 2 km apart along each of the 12 major road segments, with a total of 400 survey points (Figure S3 in Supporting Information S1). Survey results were collected with Open Data Kit (Hartung et al., 2010) and used as a cross‐reference for visual comparison against the satellite based land‐use data set from GlobeLand30. The most common land cover type in the study area was cropland and natural vegetation mosaics (Figure S4 in Supporting Information S1).

To configure the parameters in EMOD and validate the model, population data, malaria incidence, and parasite prevalence rates were collected from previous works. Population data were obtained from demographic surveys in the sugar factory command village and vicinity communities from 2018 to 2021. Clinical cases for the 2008 to 2017 period were referenced from the malaria morbidity registration books (Hawaria et al., 2019). The prevalence rate was calculated based on passive case detection implemented at the Arjo Sugar Factory Clinic and two other health facilities in 2018 and 2019. Since there were no official records of malaria control campaigns in the study area for the past 20 years, we also interviewed the district health officers, clinical staff in health facilities, and sugar factory administrations to estimate the coverage, duration, and the total number of long‐lasting insecticidal bed nets distributed and indoor residual spraying (IRS) applied (personal communication, 30 April 2022).

### 2.3 Model Approach

#### 2.3.1 Model Background

We used ParFlow‐CLM, a process‐based gridded model, to simulate hydrologic processes in the Arjo study site in Ethiopia. ParFlow solves the variably saturated subsurface flow and overland flow, while CLM calculates the canopy water balance and terrestrial energy balance, which are influenced by land cover characteristics. Due to its ability to simulate complex surface‐subsurface interactions, ParFlow‐CLM can resolve a diverse range of water bodies driven by heterogenous hydrological and geomorphological processes, which result in different breeding habitats such as rain‐fed pools, flood basins, and spring‐fed ponds (M. W. Smith et al., 2013).

EMOD was used to simulate malaria transmission in the study area. The modeled region in EMOD can be represented as a single node or divided into multiple nodes. As a stochastic ABM, it simulates the simultaneous interactions between humans and mosquitoes within each node, using decision rules based on individual agent properties with inbuilt randomness (Bill & Melinda Gates Foundation, 2023). The properties are defined by user inputs on demographic, climate, mosquito, parasite, and intervention parameters. The model simulates vector population dynamics (e.g., vector life cycle, vector survival and feeding), human population dynamics, human immunity, within‐host parasite dynamics, and effects of interventions such as antimalarial drugs and vaccines (Eckhoff, 2013). A more detailed description of ParFlow‐CLM and EMOD can be found in Texts S1 and S2 in Supporting Information S1.

#### 2.3.2 Linking Habitat Representation in EMOD With ParFlow‐CLM

In EMOD, natural larval habitats commonly comprise temporary, semi‐permanent and permanent (constant) habitats, and each habitat type is calculated based on a different equation (Eckhoff, 2011). Temporary habitats are driven mainly by rainfall and decay proportionally to evaporation rate, which is a function of temperature and humidity based on the Clausius‐Clapeyron relation.

The area of temporary habitats in each node at time t, $H_{\text{temp}}^{t}$, is calculated by:

(1) $$H_{\text{temp}}^{t}=H_{\text{temp}}^{t-1}+\lambda_{\text{temp}}P^{t}{D_{\text{cell}}}^{2}-H_{\text{temp}}^{t-1}\tau_{\text{temp}}^{t}\Delta t$$

and

(2) $$\tau_{\text{temp}}^{t}=5.1\times 10^{11}e^{\frac{-5,628.1}{T^{t}}}k_{\text{temp}}\sqrt{\frac{0.018}{2\pi RT^{t}}}\left(1-{\text{RH}}^{t}\right),$$

where $\lambda_{\mathrm{temp}}$ is a scaling factor, $P^{t}$ is rainfall at time t, $\tau_{\text{temp}}^{t}$ is a decay rate at time t, $D_{\mathrm{cell}}$ is the nodal size in degree, Δt is the time interval, $T^{t}$ is the temperature in Kelvin at time t, $k_{\mathrm{temp}}$ is a decay factor, R is the universal gas constant 8.314 J/mol/K and ${\text{RH}}^{t}$ is the relative humidity at time t.

Similar to temporary habitats, semi‐permanent habitats are also driven by rainfall, but the decay rate is a constant that is independent of temperature and humidity. Semi‐permanent habitats are configured to decay slower than temporary habitats. Using a scaling factor $\lambda_{\mathrm{semi}}$ and a decay rate $\tau_{\mathrm{semi}}$, the area of semi‐permanent habitats $H_{\text{semi}}^{t}$ is calculated as:

(3) $$H_{\text{semi}}^{t}=H_{\text{semi}}^{t-1}+\lambda_{\text{semi}}P^{t}{D_{\text{cell}}}^{2}-H_{\text{semi}}^{t-1}\tau_{\text{semi}}\Delta t.$$

Lastly, permanent habitats are assumed to be independent of rainfall, temperature, and humidity. The area, $H_{\text{perm}}^{t}$, remains the same over time and is determined by a constant scaling factor $\lambda_{\mathrm{perm}}$:

(4) $$H_{\text{perm}}^{t}=\lambda_{\text{perm}}{D_{\text{cell}}}^{2}.$$

**Figure 2:**
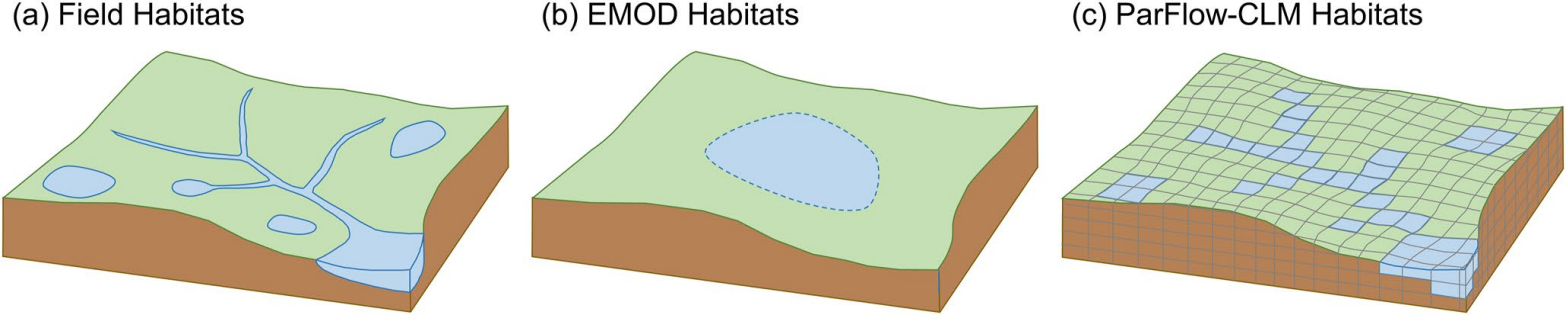
Illustration of (a) field habitats within a study area (b) conceptually lumped habitat calculated in Epidemiological MODeling within a node representing the same study area (c) spatially distributed habitats simulated in Parflow‐Community Land Model within the same node.

As shown in Figure 2, EMOD conceptually models each habitat type within a node as a lumped habitat which is oversimplified and does not have the granularity to support habitat‐based interventions. To enhance the fidelity of the model in representing habitats which are spatially distributed in reality, we simulated the habitats explicitly in ParFlow‐CLM in place of the default habitat calculation in EMOD.

ParFlow‐CLM simulated the spatiotemporal distribution of surface soil saturation, which was used to determine the availability of surface water that could contribute to ponding. While the model can generate ponding directly, it does so at a grid resolution of 100 m which is not sufficient to resolve small‐scale ponds (i.e., order of 10 m or smaller) relevant to larval habitats. Since soil saturation measures the extent to which the water content has filled up the voids within the soil, a higher soil saturation means there is a higher chance of small‐scale ponding within the 100 m grid cell. For each grid cell in ParFlow‐CLM, ponding was assumed to occur if the soil saturation exceeds a threshold, *θ*, which was calibrated based on larval habitat observations as an indication of the presence of small‐scale ponds. The duration of ponding was then quantified and referred to as Wetness Index. Based on the Wetness Index, each cell was classified into temporary (15–90 days), semi‐permanent (90–180 days) or permanent habitat (more than 180 days). Rivers with high flow rates were not considered since *Anopheles* larvae have a lower chance of surviving in fast‐moving water (Hardy et al., 2013). Details of the concept of hydrologic simulation and larval habitat identification can be found in Jiang et al. (2021). For each time step, the fraction of the study area covered by each habitat type (i.e., $F_{\text{temp}}^{t}$, $F_{\text{semi}}^{t}$, and $F_{\text{perm}}^{t}$) was calculated and input into EMOD. Finally, the area for each habitat type in each node was obtained after multiplying the fractional area coverage by the nodal area as follows:

(5) $$H_{\text{temp}}^{t}=F_{\text{temp}}^{t}{D_{\text{cell}}}^{2},$$

(6) $$H_{\text{semi}}^{t}=F_{\text{semi}}^{t}{D_{\text{cell}}}^{2},$$

and

(7) $$H_{\text{perm}}^{t}=F_{\text{perm}}^{t}{D_{\text{cell}}}^{2},$$

where $F_{\text{temp}}^{t}$, $F_{\text{semi}}^{t}$, $F_{\text{perm}}^{t}$ are the fractional area coverage of temporary, semi‐permanent, and permanent habitats, respectively.

#### 2.3.3 Habitat Larval Capacity

EMOD requires the user to define a larval capacity per unit area (LC) for each habitat type, representing the maximum hypothetical number of larvae that can co‐exist within a 1‐degree by 1‐degree habitat area. LC was then multiplied by the nodal time series habitat area in degree^2^ (Section 2.3.2). Finally, the variation in larval capacity within a node is defined.

In this study, LC was estimated using field survey data for each habitat type (Text S3 in Supporting Information S1). In Table 1, LD_dip_ represents the larval density in number of larvae per dip. We then converted LD_dip_ to an equivalent number of larvae per unit degree squared, LD, based on the opening area of the standard 350 mL mosquito larvae dipper, which is 13 cm in diameter (Orondo et al., 2023). To obtain LC, we adjusted LD by a scaling factor, *s*, during the calibration of EMOD. The adjustment is necessary because using LD directly will overestimate the larval capacity as surveyors tend to dip at locations with a higher density of larvae within a sampled habitat.

**Table 1:** Laval Density Derived From Field Survey and Calibrated Larval Capacity per Unit Area for Each Habitat Type

Habitat type	Larval density		Larval capacity per unit area
	LD_dip_ (#/dip)	LD (#/degree^2^)	LC = *s* × LD (#/degree^2^)
Temporary	0.167	1.97 × 10^11^	3.27 × 10^6^
Semi‐permanent	0.089	1.05 × 10^11^	1.74 × 10^6^
Permanent	0.440	5.18 × 10^11^	8.62 × 10^6^

**Figure 3:**
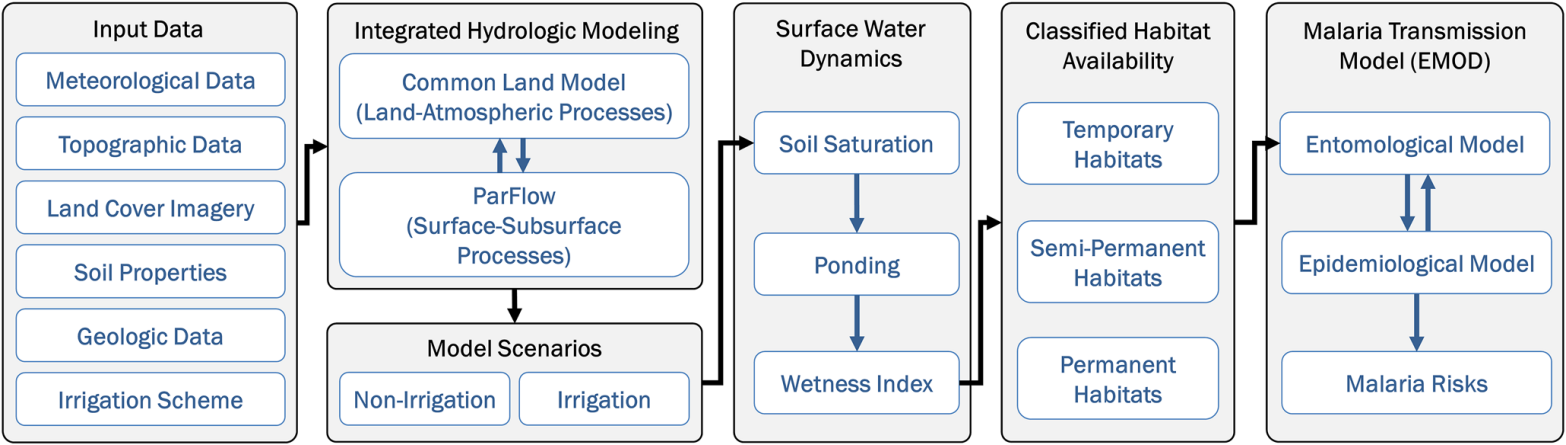
Framework for integrating ParFlow‐Community Land Model with Epidemiological MODeling (EMOD).

In summary, we identified potential larval habitats in ParFlow‐CLM and classified them into temporary, semi‐permanent, and permanent habitats as an input to the vector cycle simulation in EMOD. The overall schematic of our modeling approach is shown in Figure 3.

### 2.4 Model Configuration

#### 2.4.1 Model Domain

In a previous study, ParFlow‐CLM was successfully applied in Arjo for larval habitat identification over 1 year through 2018 (Jiang et al., 2021). Here, we expanded the hydrologic simulation to 20 years from 2000 to 2020. The model domain area was 208 km^2^ (Figure 1). To keep the computational time manageable, we decreased the spatial resolution from 50 to 100 m and reduced the number of subsurface layers from 10 to 5 layers. The thickness of the layer from ground surface to bedrock was 0.1, 0.3, 0.6, 1, and 78 m, over a total vertical depth of 80 m.

For malaria transmission modeling in EMOD, we focused on the sugarcane plantation area within the hydrologic modeling domain (Figure 1). The EMOD domain was configured as a single node measuring 10 km by 10 km.

#### 2.4.2 Model Scenarios

Three model scenarios were developed for this study. In the first scenario (*Default EMOD*), malaria transmission was simulated based on the default larval habitat equations in EMOD (Equations (1), (2), (3), (4)). In the second scenario (*Integrated EMOD or Non‐Irrigation*), the default larval habitat equations were replaced by the simulated habitats from ParFlow‐CLM through Equations (5), (6), (7), which integrates terrestrial hydrological processes. To investigate the effect of irrigation, a third scenario (*Irrigation*) was added using the same integrated approach from the second scenario, but irrigation was modeled in ParFlow‐CLM per the local schedule as described below.

**Figure 4:**
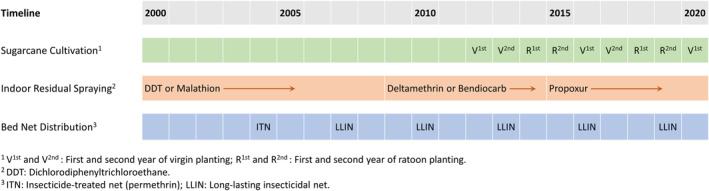
Configuration of intervention and sugarcane cultivating plan in the integrated model. The irrigation schedule can be referred to in Text S4 and Figure S6 in Supporting Information S1.

Irrigation was applied starting in 2012, and a 4‐year sugarcane planting cycle was adopted in the model, as shown in Figure 4. The cycle includes a 2‐year cycle for virgins and two 1‐year cycles for ratoons, typical of the sugarcane plantation. The irrigation scheme was designed based on the sugarcane planting cycle, and the months with irrigation are shown in Figure S6 in Supporting Information S1. During the irrigating season, irrigation occurred every 10 days for the first 3 days. Daily 5.3 mm/hr of sprinkler water was applied for 22 hr. The derivation of the irrigation rate can be found in Text S4 in Supporting Information S1.

In the three scenarios, malaria interventions, including IRS and bed net distribution, were modeled per the schedule shown in Figure 4.

The default habitat functions in EMOD are designed primarily to incorporate the variability of climate and the scaling factors and decay rates are usually calibrated to fit local data on vector abundance or entomological inoculation rate (EIR) rather than the habitat area per se (Eckhoff, 2011). This implicates other non‐habitat related factors from vector life cycle, infectivity and transmission. As our intent is to isolate the effect of incorporating hydrologic modeling on the habitat simulation, the scaling factors and decay rates should be calibrated to field data on habitat area, but it is challenging to ascertain the total area of the habitats in the study area. As such, the scaling factors ($\lambda_{\mathrm{temp}}$, $\lambda_{\mathrm{semi}}$ and $\lambda_{\mathrm{perm}}$) in *Default EMOD* were set individually such that the average area of each habitat type over the simulation period was the same as *Integrated EMOD*. In addition, the decay parameters $k_{\mathrm{temp}}$ and $\tau_{\mathrm{semi}}$ in *Default EMOD* were also adjusted to approximate the variability in *Integrated EMOD* (Text S5 in Supporting Information S1). This was to avoid the influence of parameter selection on the difference in malaria transmission results between the two scenarios.

#### 2.4.3 Model Calibration

We calibrated ParFlow‐CLM and EMOD in the irrigation scenario and used the same calibrated parameters in the *Default EMOD* and *Integrated EMOD* scenarios. This is because observed data was only available for the period after irrigation was implemented in the study area. This also prevented the effect of incorporating hydrologic modeling or irrigation from being obscured by using different parameters in each scenario.

As the spatial resolution in ParFlow‐CLM was modified from the previous study (Jiang et al., 2021), we recalibrated the saturation threshold, *θ*. The calibration was to ensure that the model will predict the occurrence of ponding at locations in line with the field surveyed larval habitats for soil saturation above the selected threshold (Text S6 in Supporting Information S1 and Jiang et al., 2021). Larval habitat data was chosen for calibration as it is a direct indication of small‐scale ponding. For the calibration of EMOD, we identified 15 key parameters (Table S3 in Supporting Information S1) after a preliminary sensitivity analysis. The calibration aimed to align the simulated prevalence rate and pattern of clinical cases with local data (Figures S9 and S10 in Supporting Information S1). The rest of the parameters were either referenced from published studies (Gerardin et al., 2015; Selvaraj et al., 2018) or set based on default values in EMOD.

#### 2.4.4 Spatial Realization of Adult Vectors Through Heterogeneity of Habitats

To demonstrate how the heterogeneity of habitats can affect distribution of adult vector abundance, we conducted an additional analysis by discretizing the study site into 100 nodes measuring 1 km by 1 km in EMOD. All the nodes were assigned the same calibrated parameters for simplicity, but the input habitat area was specific to each node. This was only applicable to the scenarios with ParFlow‐CLM integrated into EMOD. The habitat simulation in *Default EMOD* could not reflect spatial heterogeneity since it cannot process the effects of topography, soil and land cover.

## 3 Results

### 3.1 Effect of Hydrology on Larval Habitats and Malaria Transmission

**Figure 5:**
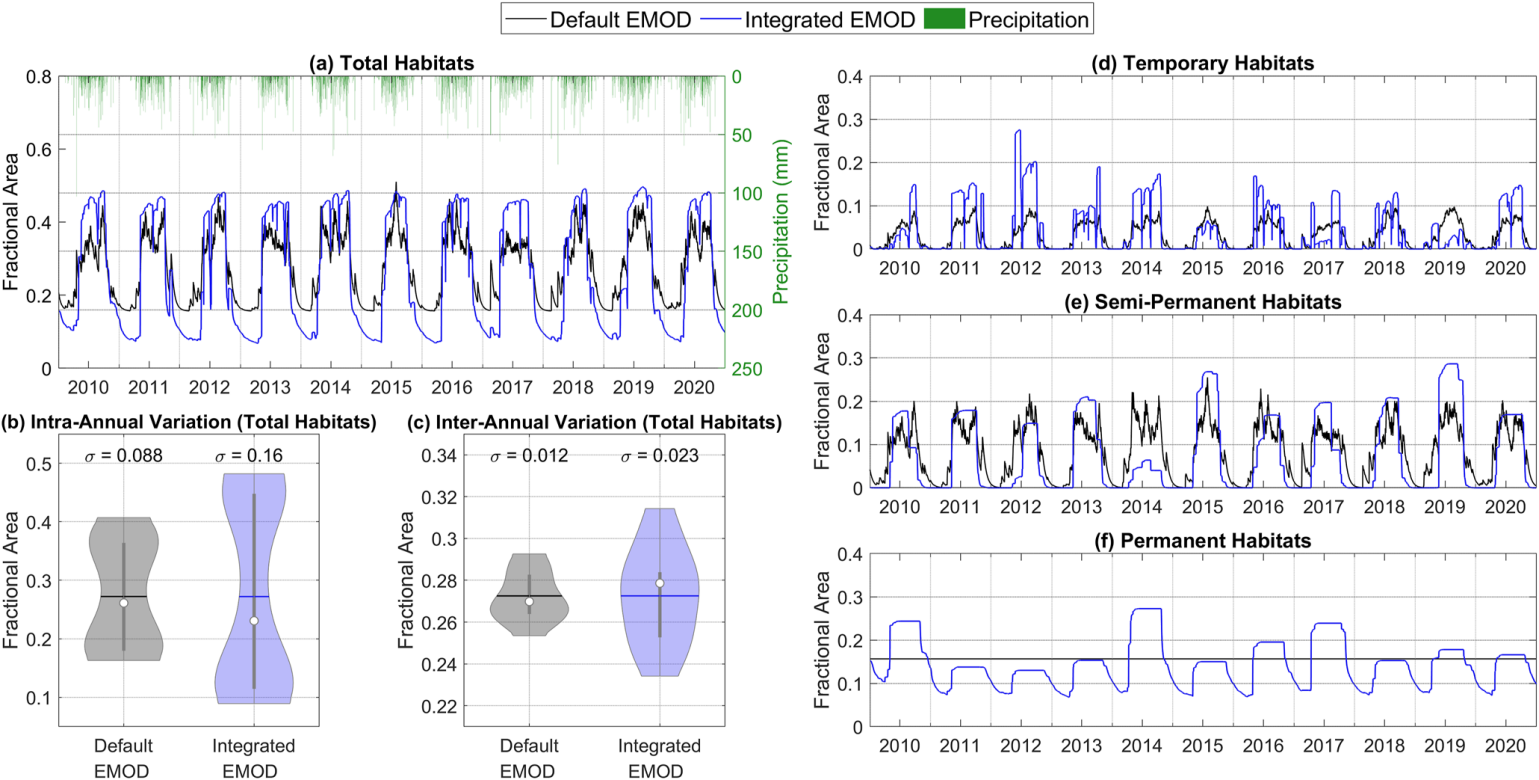
Comparison of daily simulated larval habitat area between *Default Epidemiological MODeling (EMOD)* and *Integrated EMOD*. We only show the truncated time series from 2010 to 2020 instead of the full simulation period for brevity and readability. (a) The total habitat area is broken down into (d) temporary habitats, (e) semi‐permanent habitats and (f) permanent habitats. Violin plots were used to illustrate the (b) intra‐annual distribution and (c) inter‐annual distribution of the total habitat area. Intra‐annual distribution is based on the 20‐year average habitat area for each day of a year, while inter‐annual distribution is characterized by the annual average habitat area for each year. In the violin plots, the white dot and horizontal line represent the median and mean, respectively. The vertical bar in the center of the violin plot corresponds to the interquartile range.

A comparison of the larval habitat area as a fraction of the study area between *Default EMOD* and *Integrated EMOD* is illustrated in Figure 5. In both scenarios, the total larval habitat area varied in tandem with seasonal rainfall, with a mean of 27% (Figure 5a). However, the habitats in *Integrated EMOD* exhibited less frequent daily fluctuations, with a much lower seasonal minimum and higher seasonal maximum resulting in an overall larger seasonal range than *Default EMOD*. Figure 5b shows a violin plot of the average total habitats for each day within a year (intra‐annual distribution) while Figure 5c shows a violin plot of the annual average total habitats (inter‐annual distribution) throughout the simulation period. The habitats in *Integrated EMOD* were found to have a more significant intra‐annual and inter‐annual variability as the standard deviation of the habitats is about 1.8 times and 2 times that of *Default EMOD*, respectively. The reason is attributable to the simplifying assumptions built into the habitat functions in *Default EMOD* and will be discussed further in Section 4.1.

Figures 5d–5f present the breakdown for each habitat type. Of the total larval habitat area, permanent habitats were the most dominant, accounting for 16% of the study area, followed by semi‐permanent habitats (8.4%) and temporary habitats (3.3%). In *Integrated EMOD*, the fluctuation in habitat area gradually becomes smoother from temporary to semi‐permanent and finally permanent habitats, corresponding with the increasing stability of the habitats. In each year, the distribution between the habitat types can vary significantly depending on the magnitude and duration of rainfall in that year. For example, there were more temporary habitats relative to semi‐permanent habitats in 2012 and vice versa in 2015 due to a difference in rainfall patterns.

In contrast, the difference in stability and dynamic distribution between temporary and semi‐permanent habitats was less apparent in *Default EMOD*. Notably, the area of permanent habitats remained constant throughout the years. This is a key difference from *Integrated EMOD*, in which permanent habitats were defined as habitats with more than 180 days of ponding and subject to temporal variations.

**Figure 6:**
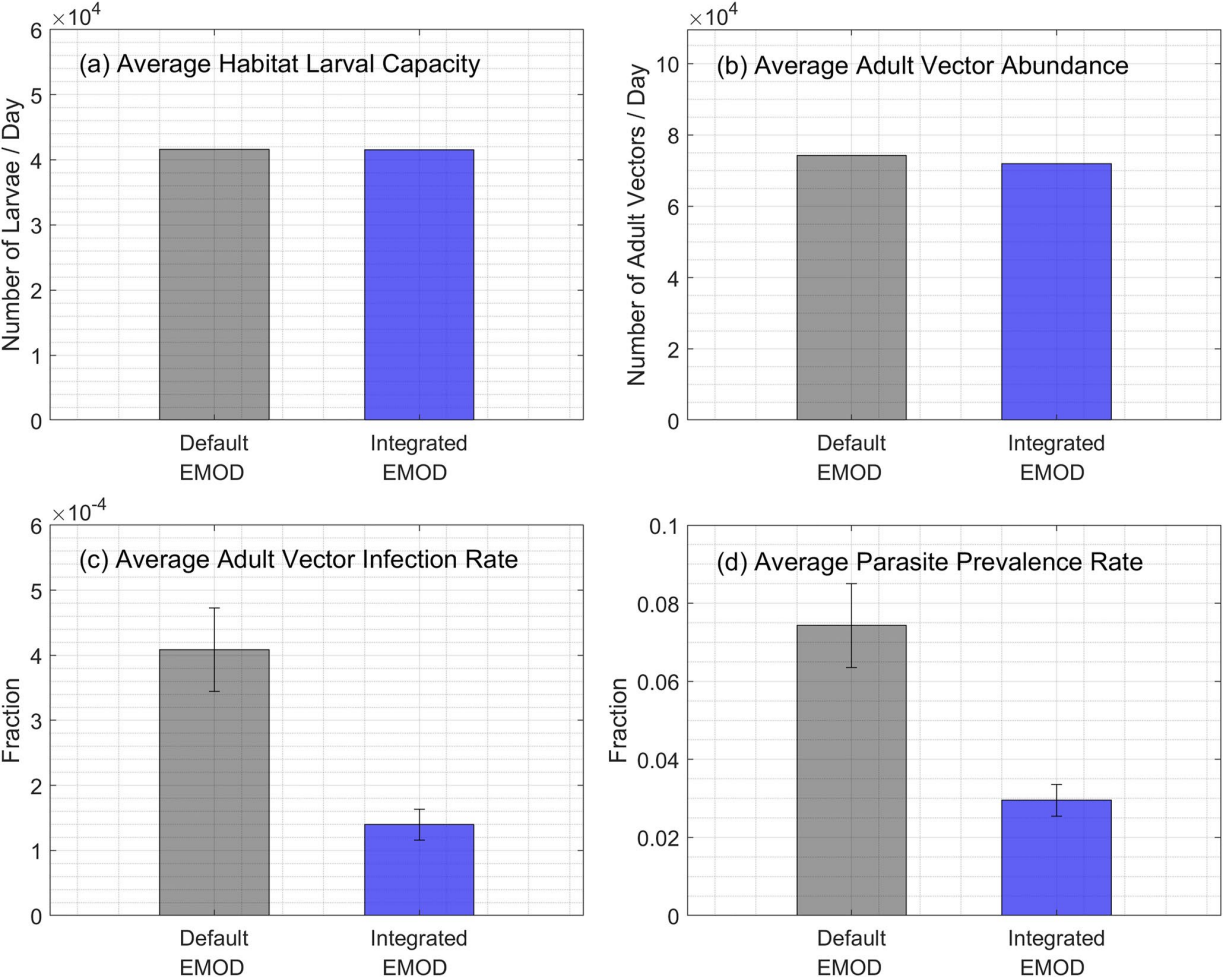
Comparison of annual average malaria transmission indicators between *Default Epidemiological MODeling (EMOD)* and *Integrated EMOD*. The indicators include (a) habitat larval capacity, (b) adult vector abundance, (c) adult vector infection rate, and (d) parasite prevalence rate.

Since the average total larval habitat area was the same in *Default EMOD* and *Integrated EMOD*, the average habitat larval capacity was identical in both scenarios (Figure 6a). However, the number of adult vectors was slightly higher (by 3%) in the *Default EMOD* scenario (Figure 6b). The resulting difference was further amplified to 2.9 times for the average vector infection rate (Figure 6c) and 2.5 times for the average prevalence rate (Figure 6d). Given that all other input data and parameters in EMOD were the same, this can be attributed to the visibly lower variability in the daily habitat larval capacity of *Default EMOD* (Figure S11c in Supporting Information S1). While the difference from *Integrated EMOD* might be small in the rainy season in some years, the larval habitat capacity is consistently much higher than *Integrated EMOD* during the dry season in all years, providing a stable environment for the vector to thrive perennially. The lower variability in habitat larval capacity is not only due to lower variability in larval habitat area but also the high larval capacity per unit area of permanent habitats whose area does not vary. In summary, the results suggest that incorporating hydrologic modeling can produce higher variability in larval habitat, resulting in lower simulated malaria transmission. To reinforce this finding, we also conducted a sensitivity analysis to test the effect of the degree of habitat variability on vector infection rate and prevalence rate (Text S7 in Supporting Information S1).

### 3.2 Effect of Irrigation on Larval Habitats and Malaria Transmission

**Figure 7:**
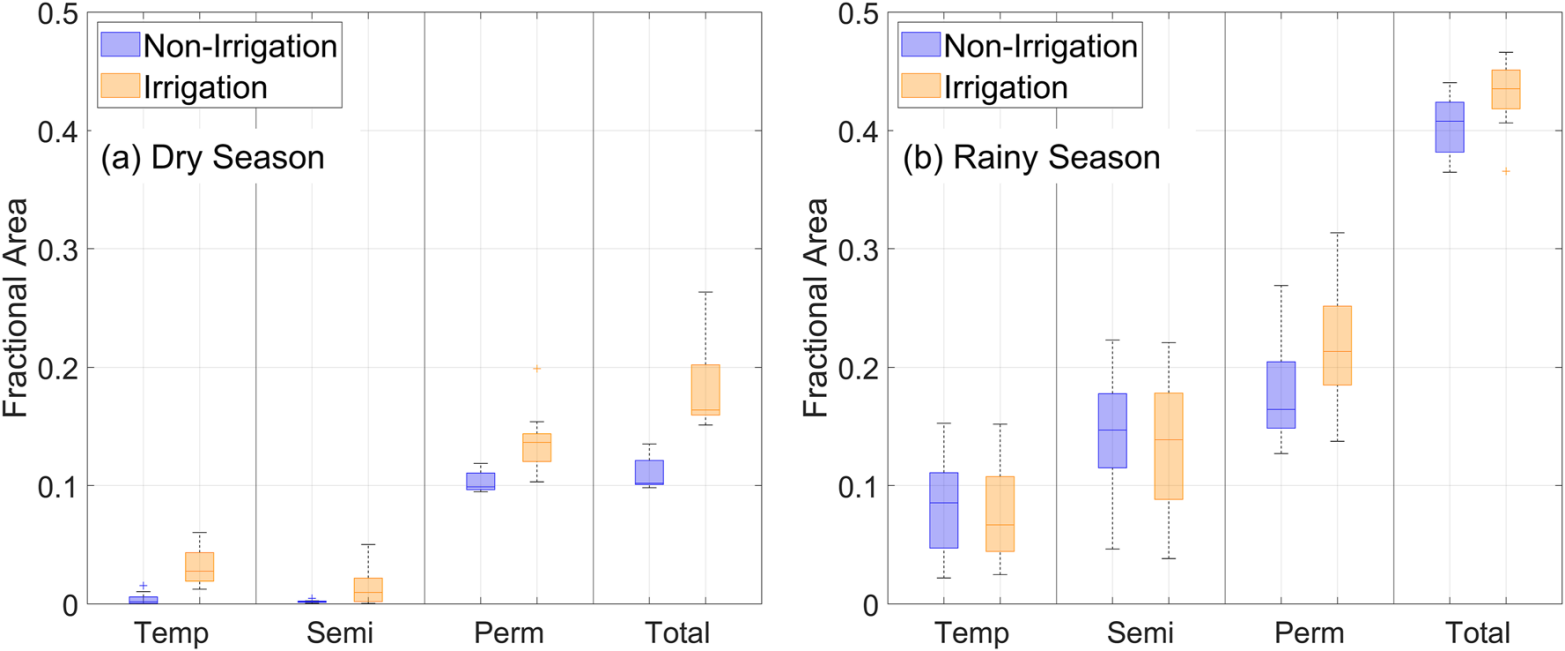
Comparison of larval habitat area during (a) dry season and (b) rainy season from 2012, the year when irrigation started, to 2020 between *Non‐Irrigation* and *Irrigation*. Habitats are further classified into temporary, semi‐permanent, and permanent types. The horizontal line inside the box represents the median, and the height of the box corresponds to the interquartile range.

Irrigation generally increased the habitat area in both dry and rainy seasons. In the dry season (Figure 7a), the increase in median fractional area was the highest for temporary habitats (7.4 times), followed by semi‐permanent habitats (6.6 times) and permanent habitats (1.3 times). Although irrigation was only applied in the dry season, it prolonged the stability of temporary and semi‐permanent habitats and converted them to permanent habitats in the rainy season (Figure 7b). For example, some of the swamps would only have existed during the rainy season if not for the additional irrigation water supplied during the dry season. Temporary and semi‐permanent habitats decreased in coverage by about 7% and 8%, whereas permanent habitats grew by 24%. From the results, habitats arising from irrigation may enable the development of vectors in the dry season while stabilizing the growth in rainy seasons.

**Figure 8:**
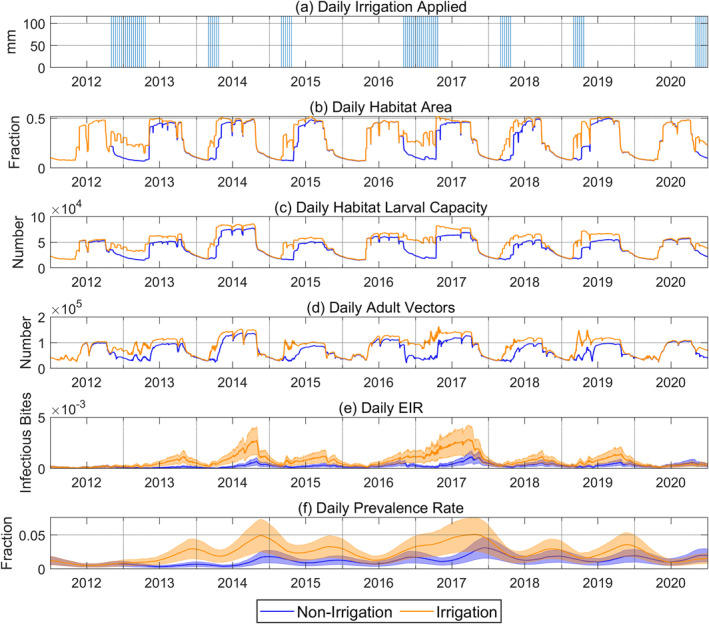
Time series of daily applied irrigation in *Irrigation* and comparison of simulated daily malaria transmission results between *Non‐Irrigation* and *Irrigation* from 2012 onwards, when irrigation started. Malaria transmission results include (b) habitat area, (c) habitat larval capacity, (d) adult vector abundance, (e) entomological inoculation rate, and (f) parasite prevalence rate.

Next, we presented the simulated times series of habitat area, larval capacity, adult vector population, EIR, and parasite prevalence for *Non‐Irrigation* and *Irrigation* over the irrigated period in Figure 8. Comparing Figures 8b and 8c, the differences in the larval capacity per unit area for each habitat type introduce more inter‐annual variability to larval capacity as the relative abundance of each habitat type is dynamic. The adult vector population's pattern generally follows habitat larval capacity. However, the EIR cycle lags the adult vector population cycle by 2 months, and the parasite prevalence cycle lags the EIR cycle by another 1 month.

The increase in habitat area arising from the applied irrigation contributed to an increase in adult vector population beyond the irrigation periods as well as EIR and parasite prevalence. The simulated daily EIR hit a maximum of 0.0029 in September 2017, a 3‐fold increase compared to *Non‐Irrigation*, due to the longest preceding irrigation period. In the same year, the maximum prevalence occurred in October, with a 1.9‐fold increase to 0.0509. It was also found that the EIR and prevalence peaks, which occurred around October/November and November/December, respectively, were shifted forward by about 1 month after irrigation was applied.

### 3.3 Spatial Variation of Vector Abundance

**Figure 9:**
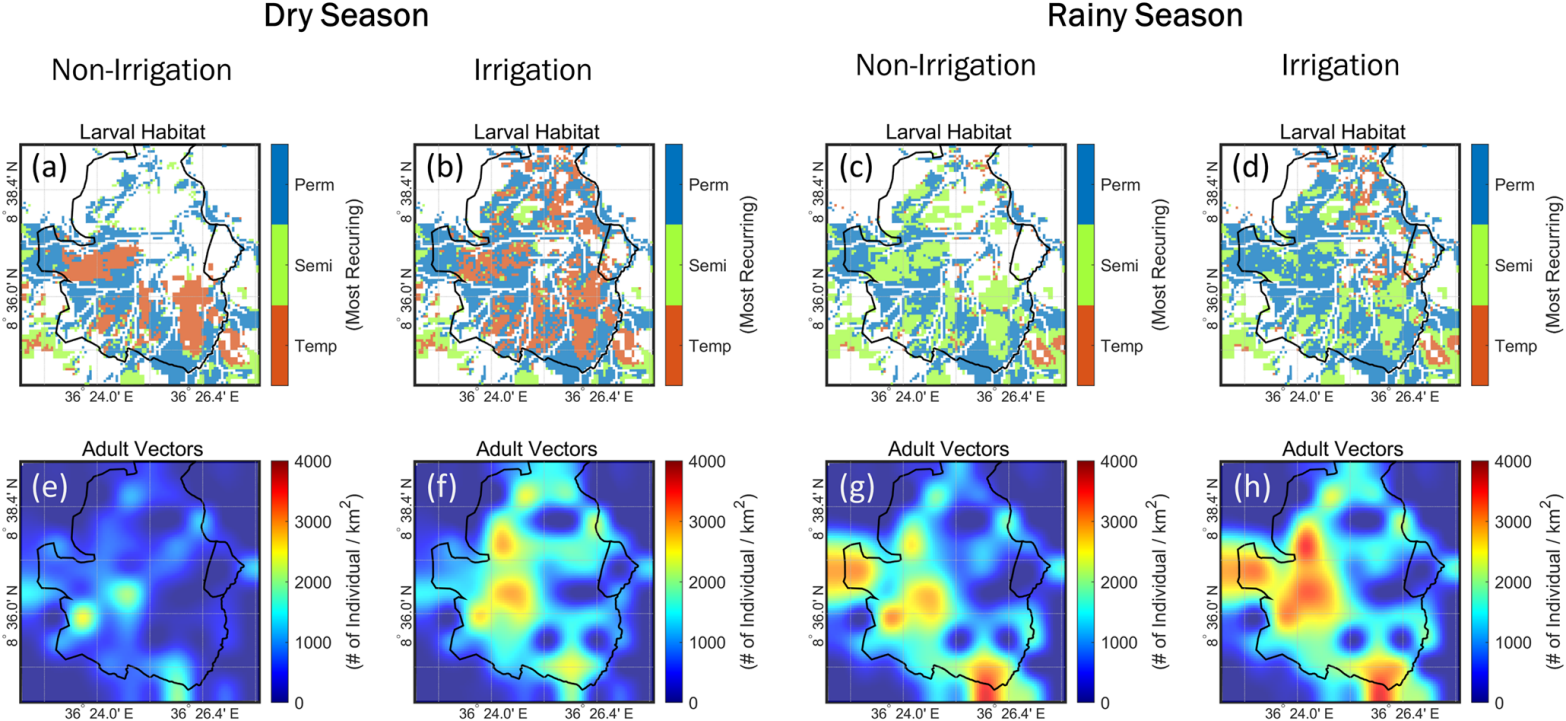
Spatial distribution of daily average larval habitats and adult vectors in the dry season (November 2016 to April 2017) and the rainy season (May 2017 to October 2017). This period was selected because the effect of irrigation on malaria transmission was the most pronounced. The simulated larval habitats and adult vectors from *Non‐Irrigation* are presented in (a) and (e) for the dry season and (c) and (g) for the rainy season. Similarly, the simulated larval habitats and adult vectors from *Irrigation* are presented in (b) and (f) for the dry season and (d) and (h) for the rainy season. Their spatially averaged values can be found in Table S4 in Supporting Information S1.

The effect of the spatial distribution of larval habitats on vector abundance is illustrated in Figure 9. Larval habitats formed more easily in the southwestern region (Figures 9a–9d) which is characterized by clay‐rich soil with low permeability (Figure S12 in Supporting Information S1). Besides soil type, the distribution of the habitat types within the study area varied substantially with hydrologic processes depending on the local topography, land use and irrigation. In both dry and rainy seasons, irrigation expanded the area covered by habitats and increased the stability of existing habitats.

Like habitat areas, the adult vector hotspots in both seasons were enlarged and intensified by irrigation (Figures 9e–9h). The increase in vector population was more significant in the dry season (Table S4 in Supporting Information S1) due to the creation of more habitats by irrigation. The adult vector hotspots were mainly concentrated around permanent habitats configured with the highest larval capacity based on field data.

## 4 Discussion

### 4.1 Role of Hydrology in Habitat Seasonality and Implications on Transmission

The average vector infection and prevalence rates in *Default EMOD* over the simulation period were significantly higher than *Integrated EMOD*. From Figure 5a, one of the most noticeable differences between the two scenarios was the degree of seasonality of the larval habitat. Although the mean habitat area was set the same in both scenarios, the magnitude of the seasonal variation was larger when hydrologic modeling was incorporated. This is mainly because the hydrologic model in *Integrated EMOD* considers complex physical processes and characteristics specific to the study area, including topography, land use, and soil. At the same time, *Default EMOD* adopts a parsimonious, one‐size‐fits‐all approach. Specifically, the default habitat function in EMOD assumed that the permanent habitat area was at equilibrium and remained constant throughout the simulation. In reality, permanent habitats such as those on river edges can vary in the area with climate conditions. In addition, there was no infiltration mechanism for the other two habitat types (Equations 1 and 3), so new ponds started forming immediately at the onset of the rainy season and continued forming toward the end of the rainy season whenever there was rainfall. The result was an earlier rising limb and a delayed falling limb in the time series compared to *Integrated EMOD* (see Figure S13 in Supporting Information S1 for an example). As the mean area in both scenarios was the same, the *Default EMOD* time series naturally ended up with a broader but flatter crest.

The association between higher habitat variability and lower prevalence in *Integrated EMOD* can possibly be explained by the vector‐borne nature of malaria parasite. During the period when there are fewer habitats, adult vector abundance is minimal and the paucity of vectors to transmit the parasite suppresses the infectious reservoir in humans. This has a cascading effect on the peak habitat area season where adult vector abundance is high, but the vector is less likely to be infected due to low gametocyte availability. Even if the human infectious reservoir can be restored quickly, transmission could still be limited by the human population. Combined with the poor transmission in the low habitat area season, the resulting overall annual average prevalence becomes lower as opposed to the case where habitat area remains relatively constant throughout the year.

Given that the study area is characterized by low‐permeability clay, the disparity in the habitat variability between *Integrated EMOD* and *Default EMOD* and its impact on malaria transmission could be amplified in another area with permeable soil that is more conducive for infiltration, where delayed pond generation becomes even more prominent. In addition, the groundwater table happens to be low in the study area so the effects of groundwater seepage in sustaining breeding habitats are less obvious. Groundwater seepage can be an important source of larval habitats in other areas, but it is not considered in *Default EMOD*. For example, larval habitats are commonly found in the vicinity of reservoirs where the groundwater level is high and groundwater seeps easily into depressions to form habitats (Kibret et al., 2019). These habitats tend to be more stable and independent of rainfall and *Default EMOD* will likely underestimate habitat area compared to *Integrated EMOD*.

EMOD was designed primarily to model disease transmission and guide efforts toward malaria eradication. Vector ecology in the form of simplified larval habitat equations is incorporated into the model with the primary goal of capturing the seasonal pattern of transmission but not the habitats per se. Malaria studies that use EMOD adjust mosquito lifecycle parameters to match real‐world transmission metrics, including, but not limited to, prevalence and incidence. In scenarios where field EIR data is readily available, the modeling of vector ecology may even be bypassed entirely. In the former, parameter calibration may not compensate for the simplified vector ecology representation in EMOD. In the latter, the approach is highly dependent on the availability of field data. By incorporating hydrologic modeling, we seek to improve the representation of larval habitats in EMOD as a first step toward a more robust simulation of malaria transmission. In the future, other mosquito lifecycle parameters that are seldom considered, such as mosquito emergence rates, should be calibrated. This requires field data beyond prevalence and incidence, such as habitat productivity and adult mosquito abundance.

### 4.2 Insights Provided by Modeling on the Effect of Irrigation

By coupling hydrologic modeling with EMOD, we were able to investigate the effect of irrigation on malaria by comparing two scenarios whereby irrigation was the only difference. This allows us to isolate the effect of other environmental and social variables, such as temperature, rainfall, topography, and demography, from the relationship between irrigation and malaria transmission. The significance of our approach is that it supplements past field comparative studies whereby the effect of irrigation could have been obscured by different field settings (Ijumba & Lindsay, 2001).

Our modeling elucidates a few ways in which irrigation affected malaria transmission dynamics through larval habitats. First, all three habitat types increased in the dry season, while temporary and semi‐permanent habitats were converted to permanent habitats in the rainy season. During the dry season, permanent habitats were the predominant habitat without irrigation, but irrigation significantly increased the area of the temporary and semi‐permanent habitats (Figure 7a). The result was an increased diversity of the habitats which agrees with field observations (Hawaria et al., 2019). On the other hand, permanent habitats became even more dominant in the rainy season with irrigation. The change in relative abundance and stability of the habitats may favor the growth and survival of one vector species over the other, shifting the predominant vector species in the extreme case (Bamou et al., 2018; Chaves et al., 2021; Naranjo‐Díaz et al., 2020).

Next, irrigation not only creates transmission all‐year round but also intensifies the primary transmission period associated with the rainy season in terms of EIR and prevalence rate (Figures 8e and 8f). Studies have shown that irrigation can extend malaria transmission throughout the year due to water availability for breeding in the dry season (Kibret et al., 2014). Our results show that irrigation during the dry season can also increase the stability of the habitats in the rainy season by creating high soil moisture conditions favorable for ponding before the onset of the rainy season. As habitat stability is linked to adult vector density (Ndenga et al., 2011), this caused a more significant proliferation in adult vectors during the rainy season compared to *Non‐Irrigation*. Besides a larger adult vector population in the rainy season, there could also be a carryover of parasites in the human population from the preceding dry season, resulting in a higher vector infection rate. This ripple effect has been observed in past studies investigating the link between malaria transmission season and preceding rainfall (Midekisa et al., 2015; Pascual et al., 2008). Our results suggest that irrigation can also produce the same cascading effect.

Third, the modeling revealed that peak malaria transmission was shifted forward by around 1 month in the irrigation scenario (Figures 8e and 8f). Studies in East Africa have shown that rainfall significantly correlates with malaria transmission with a lag time of 1–2 months (Loevinsohn, 1994; Zhou et al., 2004). The lag can be attributed to the time for infiltration to occur, runoff to accumulate in low‐lying areas, and the development time for parasite growth. In addition, past observations have proven that irrigation plays the same function as rainfall in providing larval habitats to support vector growth (Herrel et al., 2001; Ohta & Kaga, 2014). Hence, irrigation in the dry season in our study created a pseudo‐early rainy season, which causes earlier onset of mosquito breeding and an extended malaria transmission. This information is critical for the timing of malaria intervention such as IRS, which is usually applied annually. The WHO recommends the application of IRS just before the onset of the peak transmission season so to achieve the most optimal outcome (World Health Organization, 2015), the implementation would have to be timed earlier for households in an irrigated region compared to households outside of the region. This ensures fresh deposits of insecticides during the appropriate period of peak vector abundance for each region.

Lastly, we demonstrated the effect of irrigation on the spatiotemporal distribution of adult vector abundance by considering the heterogeneity of larval habitats (Figure 9). The results presented were derived based on a sprinkler‐irrigated sugarcane plantation but the effect of irrigation can vary with crop type and irrigation practice (Jaleta et al., 2013; Mboera et al., 2010). Studies in Tanzania found the highest vector abundance in rice fields compared to sugarcane plantations and maize farms (Ijumba et al., 2002; Mboera et al., 2010). Rice has an intense water demand that is often met by flooded irrigation which forms permanent water bodies independent of rainfall, making seasonal variation of vector abundance less obvious. Sugarcane also requires a large water supply but is susceptible to waterlogging so sprinkler irrigation or gravity‐fed irrigation canals are often used over flooded irrigation, resulting in fewer permanent habitats. Lastly, maize is usually cultivated in rainfed conditions whereby habitats are minimal and more seasonal compared to rice fields. Separately, adjusting the irrigation interval can also affect vector abundance through its influence on habitat stability. For instance, intermittent irrigation has been found to be effective in reducing larvae population compared to continuous irrigation by preventing the development of larvae into late‐stage immatures (Chan et al., 2022; Djègbè et al., 2020).

While past observations have told us that irrigation can increase the adult vector population (Demissew et al., 2020), it remains a challenge to predict where and when breeding will occur (Frake et al., 2020). Integrating local irrigation practices and environmental characteristics such as land use, topography, and soil properties, our model provides new insights into the breeding hotspots broken down into temporary, semi‐permanent, and permanent habitat types. This can help larval source management (LSM) as a supplementary vector control by prioritizing resources for operational planning. LSM is known to be efficient where habitats are findable, few and fixed (Djamouko‐Djonkam et al., 2019; Stanton et al., 2021). Based on the results, we can identify the location of habitats, determine the period with manageable habitat abundance, and single out semi‐permanent and permanent habitats for targeted larviciding or habitat removal. Larviciding is achieved in EMOD by reducing the larvae population with a waning effect whereas habitat removal can either be implemented by adjusting the local drainage property in ParFlow‐CLM or excluding the area when the fractional habitat area coverage is passed from ParFlow‐CLM to EMOD. Besides LSM, comparing *Non‐Irrigation* to *Irrigation* also allows us to distinguish habitat hotspots induced by irrigation from those already present without irrigation. Other means, such as water resource management, can then be considered to control the former.

### 4.3 Limitations

By incorporating hydrologic modeling into EMOD, we demonstrated the effect of irrigation on malaria transmission and showcased the capability of the framework in resolving larval habitats and adult vector abundance spatially. However, a few simplifying assumptions were made in this study which could have introduced uncertainties in the model results.

First, ponding was derived from soil saturation and a calibrated saturation threshold. This was necessitated by the fact that the model resolution was still significantly coarser than that of the habitats due to computational limits. As such, the larval habitat capacity was calibrated to avoid overestimation of the simulated adult vector population. In calibrating the saturation threshold, we used field surveyed larval habitats as an indicator of ponding locations but did not check for false positives in the simulation of ponding. This is because we did not have any data of locations without ponding and it is also challenging to categorically rule out small scale ponding within a 100 m grid cell of the hydrologic model. One possible solution to address this scale mismatch is to downscale soil saturation to a finer resolution using a scaling relationship derived from topographic data (Le et al., 2019).

Second, a simplified irrigation scheme was implemented. Although we tried to replicate the irrigation design parameters as much as possible, we still had to make a few assumptions. For example, the irrigation application depth was estimated based on an assumed root depth of 2 m. In reality, sugarcane can reach soil depths up to 6 m (D. W. Smith et al., 2005) so the amount of irrigation water could have been underestimated, possibly causing fewer larval habitats to form. We also assumed that sprinkler irrigation was applied for 3 days each time in order to match the design irrigation rate of ∼5 mm/hr in the local report (see Text S4 in Supporting Information S1). The duration of each irrigation event may vary from 1 to 3 days in practice (Kassing et al., 2020). For the same quantity of irrigation applied, a shorter irrigation duration will result in a higher irrigation rate. If the irrigation rate exceeds the infiltration rate, runoff occurs resulting in lower water use efficiency and higher possibility of ponding. In this case, we would have underestimated the larval habitat area.

Lastly, in deriving the spatial distribution of adult vectors from the larval habitats, except for the habitat area, each node was configured with the same parameters including human population. Although larval habitat availability is an essential predictor for adult vector emergence, the spatial distribution of the human host is also crucial in influencing the movement patterns of adult vectors as a female mosquito needs to feed on human blood before it can lay eggs. Given that the human settlements were only concentrated in certain locations in the study area, it is likely that the habitats in the vicinity will have a higher contribution to transmission. This was not factored into our study which focuses more on advancing habitat representation in a malaria model. Currently, the malaria model in EMOD is set up such that each node has to be defined with a non‐zero human population which does not allow a habitat to be simulated on its own as a node. In future, improving the versatility of the nodal configuration and incorporating host‐seeking and oviposition habitat‐foraging behaviors into adult vector movement in EMOD will enable the proposed framework to fully take advantage of the spatial capability of hydrologic modeling.

## 5 Conclusion

Malaria transmission is intrinsically related to larval habitats, which cannot be characterized by climate alone. By coupling a hydrologic model with an agent‐based malaria model, the variability of larval habitats increased and resulted in significantly lower malaria transmission as opposed to modeling habitats based on a simplified function of climate factors. We also demonstrated how habitat heterogeneity based on hydrologic processes could affect the spatiotemporal distribution of adult vector abundance.

The hydrology‐integrated framework enabled us to investigate the effect of irrigation on malaria transmission through changes to larval habitats broken down into temporary, semi‐permanent, and permanent types. The results indicated that all three habitat types increased in the dry season, while temporary and semi‐permanent habitats were converted to permanent habitats during the rainy season. This influenced the transmission dynamics significantly as the transmission was sustained all‐year round and intensified during the primary season. Lastly, the peak malaria transmission was found to be shifted forward by around 1 month. These insights can help guide malaria intervention strategies to mitigate the effect of irrigation.

The study presents a novel generalizable framework that simulates the spatiotemporal dynamics of malaria transmission under the influence of irrigation by integrating hydrologic modeling with an ABM. The framework is a first step toward developing tailor‐made intervention strategies by simulating different water resource management practices. This is crucial to the continued implementation of irrigation schemes for food security while minimizing the impact on malaria transmission.

## Conflict of Interest

The authors declare no conflicts of interest relevant to this study.

## Supporting information

Supporting Information S1

## Data Availability

The simulation softwares used in this research, ParFlow‐CLM and EMOD, are available at Zenodo (S. Smith et al., 2023) and Github (Schripsema et al., 2019) respectively. The precipitation records used as model input can be downloaded from the Data Portal at Center for Hydrometeorology & Remote Sensing (Nguyen et al., 2019). Other climate input data can be retrieved from Climate Data Store (Hersbach et al., 2023). Surface elevation data can be purchased through the AW3D website (NTT DATA CORPORATION & ESTEC, 2023). Land cover data are available from the supplement section of Chen et al. (2015) and soil data can be downloaded from the SoilGrid database (ISRIC—World Soil Information, 2023). The larval habitat and malaria incidence data used to calibrate EMOD are available from Zenodo (Jiang, Lee, et al., 2023).
