# Supplementary material for: Investigating the Impact of Irrigation on Malaria Vector Larval Habitats and Transmission Using a Hydrology‐Based Model
Source: Geohealth. 2023 Dec 10;7(12):e2023GH000868. doi: 10.1029/2023GH000868 (PMC10711417; doi:10.1029/2023GH000868)
Supplement: Supplementary file 1 — Supporting Information S1 [file GH2-7-e2023GH000868-s001.pdf]

AGU PUBLICATIONS

GeoHealth

Supporting Information for

Investigating the Impact of Irrigation on Malaria Larval Habitats and Transmission Using a Hydrology-Based Model

Ai-Ling Jiang[1], Ming-Chieh Lee[2], Prashanth Selvaraj[3], Teshome Degefa[4, 5], Hallelujah Getachew[4,5,6], Hailu Merga[7], Delenasaw Yewhalaw[4,5], Guiyun Yan[2], Kuolin Hsu[1]

[1] Center for Hydrometeorology and Remote Sensing, Department of Civil and Environmental Engineering, University of California Irvine, Irvine, CA, USA

[2] Department of Population Health and Disease Prevention, School of Public Health, Susan and Henry Samueli College of Health Sciences, University of California Irvine, Irvine, CA, USA

[3] Institute for Disease Modeling, Bill and Melinda Gates Foundation, Seattle, WA, USA

[4] School of Medical Laboratory Sciences, Institute of Health, Jimma University, Jimma, Ethiopia

[5] Tropical and Infectious Diseases Research Center (TIDRC), Jimma University, Jimma, Ethiopia

[6] Department of Medical Laboratory Technology, Arbaminch College of Health Sciences, Arba Minch, Ethiopia

[7] Department of Epidemiology, Institute of Health, Jimma University, Jimma, Ethiopia

Contents of this file

Text S1 to S7
Figures S1 to S14
Tables S1 to S5

Introduction

This file contains additional information on data collection, model development, calibration and simulation results.

Text S1. ParFlow-CLM Overview

ParFlow-CLM is a physical-based distributed model that couples an integrated hydrologic model, ParFlow, with a land surface model, Common Land Model (CLM). ParFlow simulates surface and subsurface flow in an integrated fashon by simultaneously solving Richards equation and shallow water equations (Kuffour et al., 2020) in three spatial dimensions. This is done by using Richard's equation to simulate variably saturated subsurface flow with a free surface overland flow boundary condition to accurately account for the surface-subsurface interactions (Kollet & Maxwell, 2006). The overland flow boundary condition can be activated and deactivated whenever the surface layer becomes saturated or unsaturated. Both saturation excess and infiltration excess mechanisms are allowed and streams occur naturally without the need to predefine river locations. In this study, the chosen boundary condition combines the diffusive wave approximation of the shallow water equations and Mannings equation. All fluxes simulated In ParFlow are driven by gradients in the hydraulic head.

CLM solves the land-water-energy balance, which includes evaporation, transpiration, snow processes, heat fluxes, and radiation partitioning (Maxwell & Miller, 2005). The two models can be connected across user-defined soil layers. Evapotranspiration and root uptake fluxes are calculated by CLM and incorporated in ParFlow as water fluxes into and out of the model through source or sink terms in Richards equation for subsurface flow. The fluxes are influenced by land surface and vegetation type which is characterized by a suite of parameters including soil color, canopy roughness, and green leaf area.

More details on ParFlow-CLM can be found in Kuffour et al. (2020).

Text S2. EMOD Malaria Model Overview

EMOD is an agent-based, mechanistic model which tracks interacting individuals in the form of humans and vectors and their environment to simulate malaria transmission at the population level (Bershteyn et al., 2018). This differs from a compartmental model which classifies humans and vectors into different homogenous population groups referred to as compartments and tracks the population based on a system of differential equations.

In EMOD each simulated individual goes through the various infection states of the susceptible-exposed-infected-recovered-susceptible epidemiological model (SEIRS). During each discrete 1-day time step of the simulation, the vector population evolves through maturation, mating, feeding and death within the model domain referred to as a node. Successful feeding results in human infection and in turn, the malaria parasite is transmitted from the infectious human to the next susceptible vector. The node can be configured to represent any desired geographic scale ranging from the household level to the country level and the simulation can also include multiple nodes, with migration of both vector and human population enabled between nodes (Eckhoff, 2011; Eckhoff & Wenger, 2016).

The model is built upon six components namely, climate, larval habitat, vector transmission model, malaria infection and immune model, malaria symptoms and diagnostics and intervention (Bill & Melinda Gates Foundation, 2023). Climate can be configured for each node based on pre-set classification system tied to the native vegetation or user uploaded data. In our case, we used climate data sourced from PERSIANN CCS-CDR (Sadeghi et al., 2021). In the

default EMOD habitat model, there are temporary, semi-permanent and permanent natural habitats which are influenced by climate through rainfall and temperature and human-driven habitats which are a function of human population. The larval habitat area determines the number of eggs that develop into larvae as well as the number of emerging adult vectors. In the vector transmission model, the vector population is tracked throughout the mosquito life-cycle from egg to larvae and finally adult, which then progresses to the feeding cycle until death, comprising host-seeking, feeding, and egg-laying (Bill & Melinda Gates Foundation, 2023a, 2023b). The malaria infection and immune model tracks the parasite count for each infected individual, taking into account the immune response to infection. The model also tracks malaria symptoms such as fever and anemia and parasite count from diagnostics test to evaluate disease severity and mortality (Bill & Melinda Gates Foundation, 2023c). A clinical case is established when fever surpasses a certain threshold. Lastly, the intervention component allows the effect of antimalarial drugs, larviciding, long-lasting insecticidal nets (LLINs) and indoor residual spraying (IRS) to be modelled. For example, users can configure larviciding in the model based on a larval killing efficacy which is then applied on the larvae population through a decay function.

Text S3. Larval density estimation

In the study area, the surveyed larval habitats include drainage ditch, river edge/reservoir shoreline, swamp/marsh, rice puddle, animal footprint, tire track/road puddle, man-made pond, natural pond/rain pool, rock pool, water container, irrigation canal, and brick pit. The larval habitats were classified as temporary, semi-permanent, or permanent based on their natural characteristics. Since larval density can be significantly different in the dry and rainy seasons (Hinne et al., 2021; Kweka et al., 2012) and the timing and duration of the survey periods were inconsistent, we sorted the measured larval densities from the 769 sample points (Figure 1) into the dry season (January to April; November to December) and the rainy season (May to October). We then calculated the average larval density for each season as shown in Figure S4.

In the surveyed area, the larval density for temporary habitats was higher in the rainy season than in the dry season during which the habitats are less stable. On the other hand, the larval densities for semi-permanent and permanent habitats were higher in the dry season. Most of the semi-permanent and permanent habitats were associated with river edges and swamps, whereby the larvae are prone to flushing in the rainy season. Finally, the larval density in Table 1 was calculated based on the average dry season and rainy season densities.

Text S4. Irrigation schedule design

Figure S6 shows the monthly irrigation schedule obtained from the Arjo-Didessa Sugar Factory, which is tailored to the sugarcane planting cycle.

To model irrigation in ParFlow-CLM, the irrigation interval and rate are required user inputs. A design report provided by the factory recommended 8-12 days for the design of the local irrigation system, so we set the irrigation interval as 10 days.

To determine the irrigation rate, we first calculated the irrigation depth, defined as the amount of water that needs to be applied when the soil water content is depleted to the wilting point. The irrigation depth ($IrrD$) was calculated as

$$IrrD = (FC - WP) \times Depth_{soil}, \quad \text{(S1)}$$

where FC is the field capacity, WP is the permanent wilting point and $Depth_{soil}$ is the soil depth.

The study area is characterized by clay and clay loam with low permeability. Based on resources by the Northeast Region Certified Crop Advisor (Cornell University, 2010), the field capacity volumetric soil moisture content of clay was set as 50%, and the wilting point volumetric soil moisture content was set as 15%. A soil depth of 2 m was assumed. Using Equation (S1), an irrigation depth of 700 mm was obtained.

We configured the irrigation to be applied when 50% of the irrigation depth was depleted; hence, the actual irrigation depth to be applied over the 10-day irrigation interval was 350 mm. Adopting an intermittent irrigation strategy, we set the irrigation to be applied for 22 hours a day over 3 days within the 10-day cycle. Each irrigation period was assumed to be 3 days in order to match the local design irrigation rate of 5.1 mm/hr. Based on this assumption, the irrigation rate was calculated to be 5.3 mm/hr.

Text S5. Derivation of scaling factor and decay rate in the EMOD default habitat equations

In EMOD, the scaling factor (λ_{temp}, λ_{semi} and λ_{perm}) and decay rate (k_{temp} and τ_{semi}) are usually calibrated to match field observations of the total area of all larval habitats. As it is challenging to obtain this data for the study area, we used the simulated habitat area from *Integrated EMOD* to calibrate scaling factor and decay rate. As the permanent habitat area in EMOD is constant, λ_{perm} is set as the temporal mean of the fractional study area covered by permanent habitat (F_{perm}^{t}) derived from ParFlow-CLM.

For k_{temp} and τ_{semi}, the default values in EMOD are 0.05 and 0.01. We conducted a sensitivity analysis of the intra-annual and inter-annual variability of fractional habitat area for k_{temp} and τ_{semi} ranging from 0.01 to 0.2 as shown in Figure S7. For each iteration, λ_{temp} and λ_{semi} were set so that the mean temporary/semi-permanent habitat area matched ParFlow-CLM. Intra-annual variability was calculated in terms of the standard deviation of the 20-year average habitat area for each day of the year. Inter-annual variability was characterized by the standard deviation of the annual average habitat area for each year. Based on the sensitivity analysis results, k_{temp} was set as 0.07 to match both the intra-annual variability and inter-annual variability of habitats from ParFlow-CLM as closely as possible. τ_{semi} was set as 0.01 by the same logic but it also has to be smaller than k_{temp} since semi-permanent habitats should conceptually decay slower than temporary habitats.

Text S6. Model calibration

At each grid cell, ponding is assumed to occur if the soil saturation exceeds the threshold, θ. Therefore, the threshold was calibrated to ensure that the model will predict the occurrence of ponding at locations in line with the field-surveyed larval habitats for soil saturation above θ. The value for θ was obtained based on a sensitivity analysis by altering the threshold and noting the corresponding change in the probability of detection (POD). The POD

determines if the model can predict an aquatic habitat successfully and can be calculated based on the ratio of the number of successful predictions or hits, H, to the total number of samples, S:

$$POD = H/S. \tag{S2}$$

Figure S8 shows the results of the sensitivity analysis. Generally, the POD curve is higher for the simulation excluding dry season. This is because irrigation was only approximated by a simplified scheme in the dry season and may not reflect the localized irrigation dynamics. As the threshold was lowered, POD increased because ponding occurred across a larger area in the model. The influence of topography on the ponding was weakened, and the soil type became the dominant factor. On the other hand, when the threshold was increased, less ponding was predicted, resulting in a lower POD but the topographic variability was better represented. Therefore, we selected a threshold of 0.75 for a reasonable POD of 0.66 (excluding dry season) without obscuring topographic variability.

In EMOD, we calibrated 15 key parameters identified from a preliminary sensitivity analysis, and Table S3 presents the calibrated values. Using the calibrated parameters, we compared the simulated prevalence rate against field data for January 2018 and October 2018 (**Figure S9**). The results are within the same order of magnitude. In addition, we compared the simulated monthly number of clinical cases with the recorded malaria cases from April 2018 to May 2020 (Figure S10). Apart from the two peaks missed in October 2018 and November 2019, the simulated malaria cases compare reasonably well with observation in terms of magnitude and pattern. As the clinical malaria cases were sourced from major hospitals within the study area, the two peaks in recorded cases could be anomalous due to an influx of patients from outside seeking treatment at the hospitals. Overall, the model shows good agreement with the field observation.

Text S7. Habitat Seasonality and Implications on Transmission

To evaluate the effect of the degree of seasonality in the larval habitat on malaria transmission, we conducted a sensitivity analysis using a synthetic sinusoidal time series for larval habitat fractional area with the same mean but different amplitudes:

$$F_{\alpha}^{t} = \alpha \cos(365.25t + 125.2) + \bar{F}, \tag{S3}$$

where α is the amplitude of fractional area, F_{α}^{t} is the fractional area at time t, $\bar{F}$ is the mean fractional area specific to the study derived from the hydrologic model.

The sensitivity analysis results can be found in Figure S14 and are summarized in Table S5. By reducing the seasonal amplitude from 0.2 to 0.1, the adult vector population remained relatively unchanged, but the vector infection and prevalence rates tripled. For the extreme case when α was reduced to 0, the vector infection and prevalence rates increased further by 4.29 times and 4.80 times, respectively. This finding agrees with the higher simulated malaria transmission in *Default EMOD* compared to *Integrated EMOD* (Figure S11e-g). It is possible that in the case where α was 0, the consistent adult vector population arising from the invariant habitat availability resulted in a stable parasite transmission throughout the year. As α increased, the disparity between the high and low vector abundance seasons increased. In the low vector abundance season, the transmission was minimal. In the high vector abundance season,

transmission increased but would be limited by the human population. This resulted in an overall lower annual average vector infection rate and prevalence. Therefore, a nuanced approach considering the trend of the mean and the degree of the seasonality of larval habitats is required to predict malaria transmission accurately.

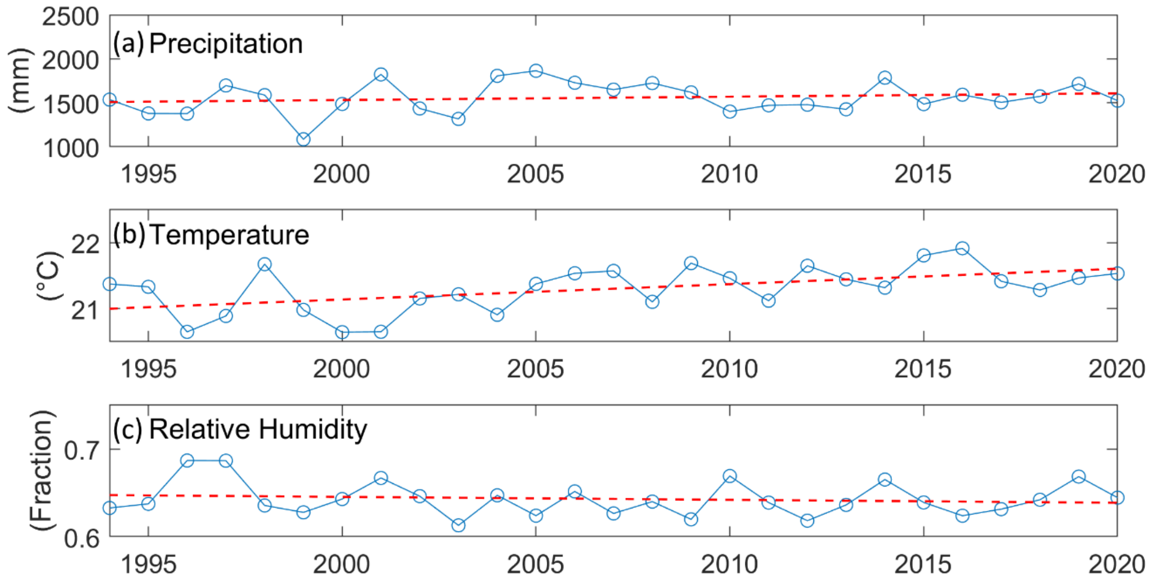

Figure S1. Annual climate data from PERSIANN-CCS-CDR and ERA5 for the study area. (a) total precipitation, (b) average temperature and (c) average relative humidity. The red dashed line represents the linear trendline in each subplot.

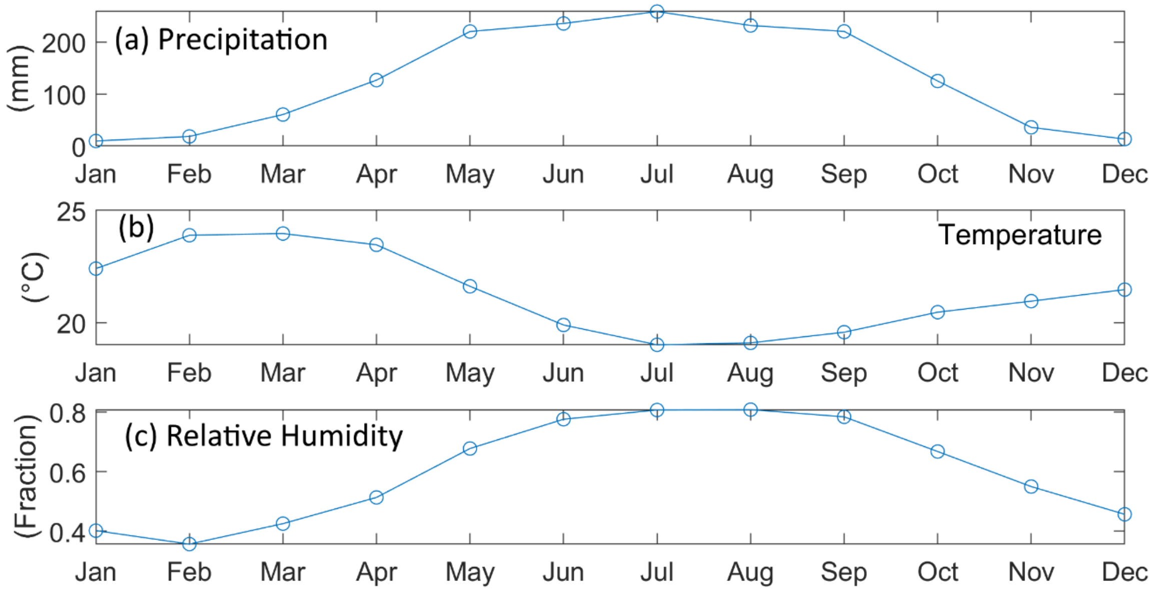

Figure S2. Monthly climate data (averaged from 1994 to 2020) derived from PERSIANN-CCS-CDR and ERA5 climate data for the study area. (a) total precipitation, (b) average temperature, and (c) average relative humidity.

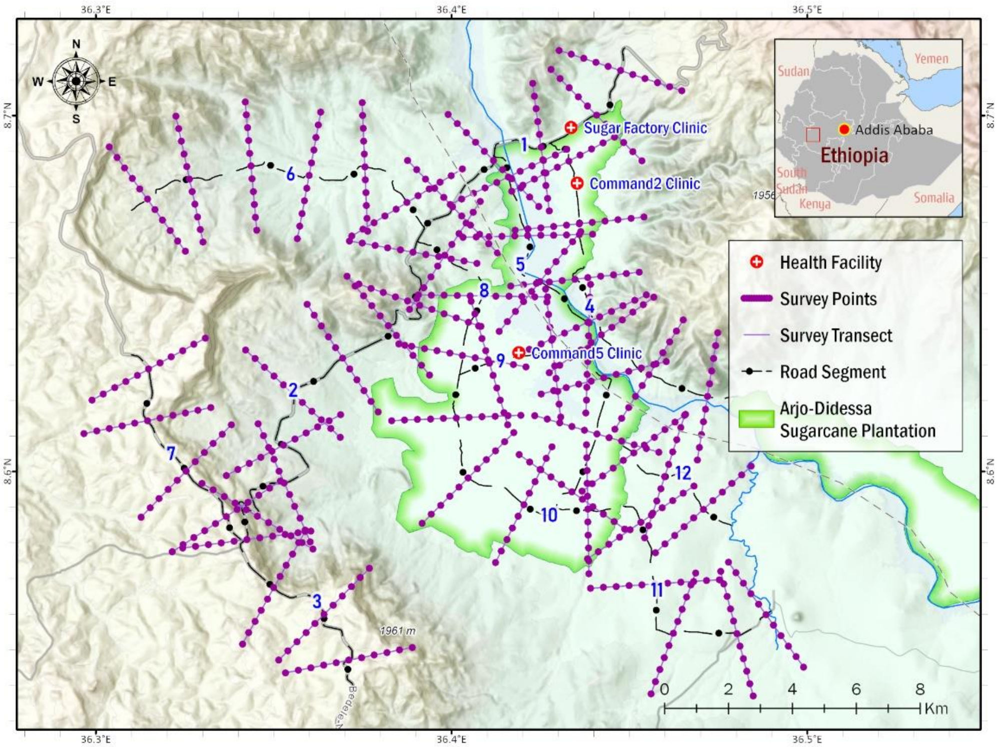

Figure S3. Land use survey locations.

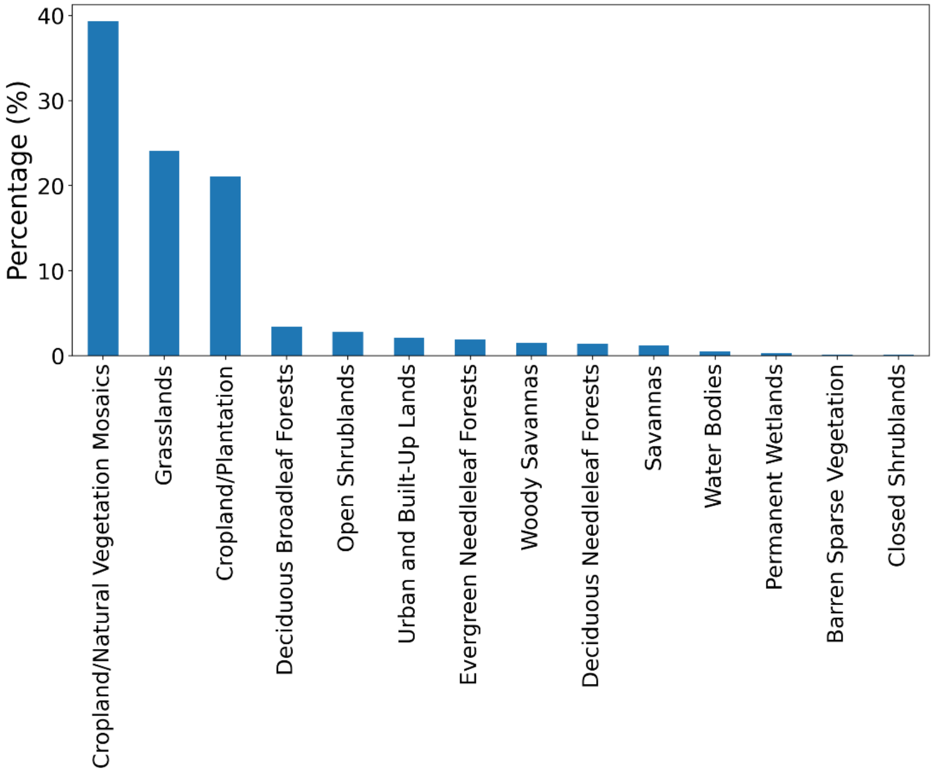

Figure S4. Percentage distribution of International Geosphere-Biosphere Programme type from land use survey.

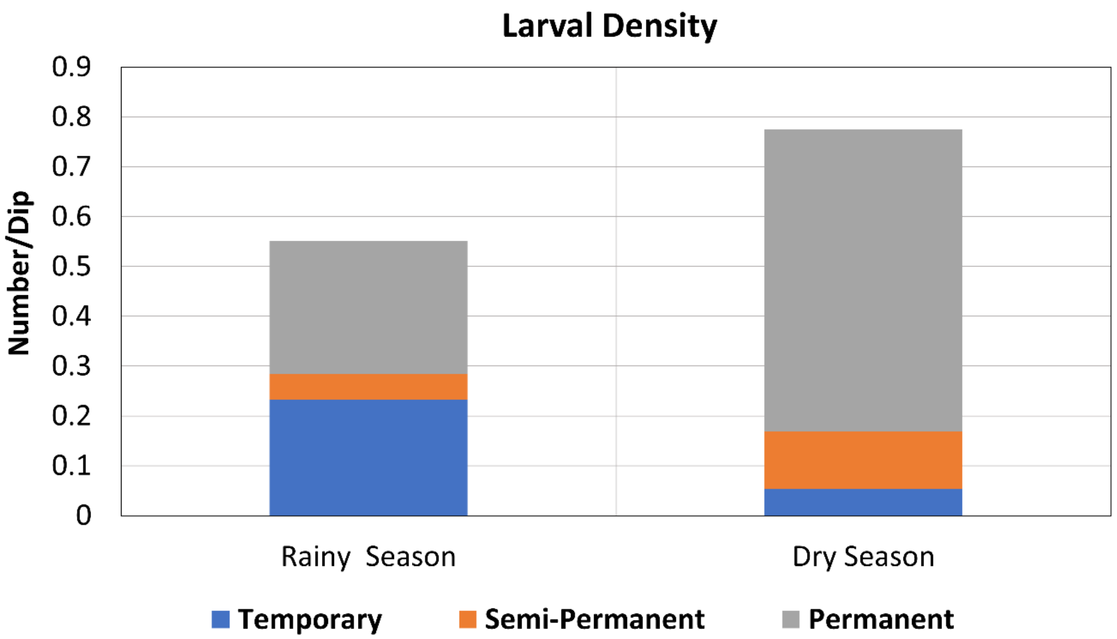

Figure S5. Average larval densities for temporary, semi-permanent, and permanent habitats during rainy and dry seasons from field survey.

(a)	1st Year (Virgin Planting)												2nd Year (Virgin Planting)												1st Ratoon			
Mn	1	2	3	4	5	6	7	8	9	10	11	12	1	2	3	4	5	6	7	8	9	10	11	12	1	2	3	4
Sym.	MA	H	H	LW	P	P	P	P	RF	RF	IR	IR	IR	IR	IR	IR	RF	RF	RF	RF	RF	RF	MA	MA	MA	H	IR	IR

(b)	1st Ratoon												2nd Ratoon												Virgin Planting			
Mn	1	2	3	4	5	6	7	8	9	10	11	12	1	2	3	4	5	6	7	8	9	10	11	12	1	2	3	4
Sym.	MA	H	IR	IR	RF	RF	RF	RF	RF	RF	MA	MA	MA	H	IR	IR	RF	RF	RF	RF	RF	RF	MA	MA	MA	H	H	LW

Figure S6. Arjo-Didessa Sugar Factory sugarcane plantation irrigation schedule. A typical sugar planting schedule includes (a) a 2-year cycle for virgin planting and (b) a 1-year cycle for the following 2 ratoons. MA: Maturity/water drain; H: Harvesting; LW: Land work; P: Planting; RF: Rainfed; IR: Irrigation.

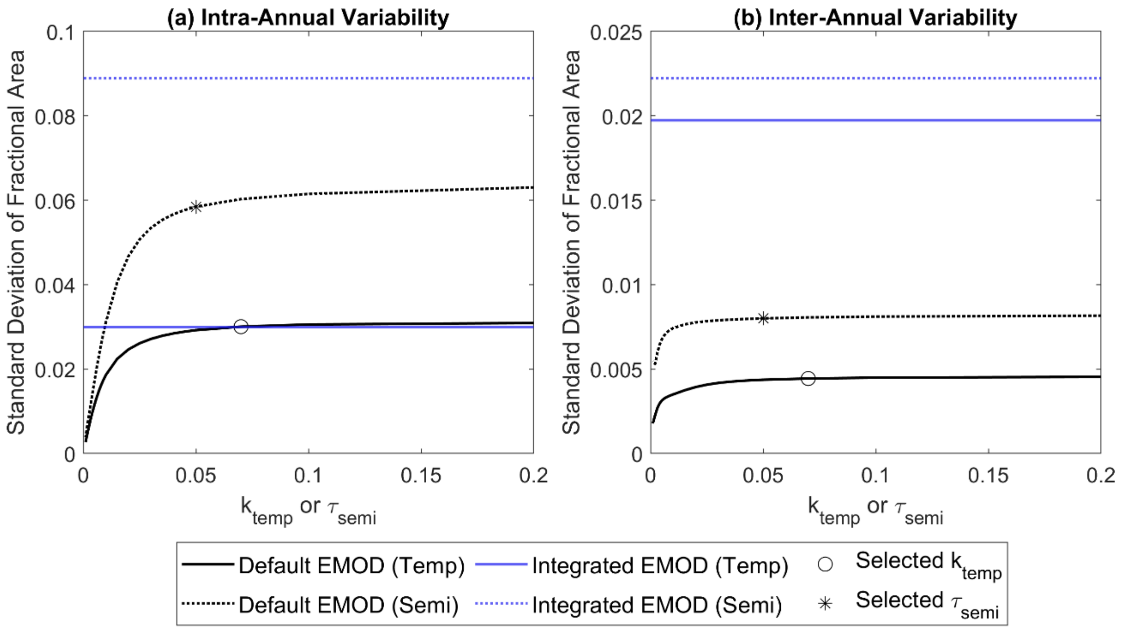

Figure S7. Adjustment of decay factors for temporary (k_{temp}) and semi-permanent habitats (τ_{semi}) in default EMOD function based on sensitivity of (a) intra-annual variability and (b) inter-annual variability of habitats.

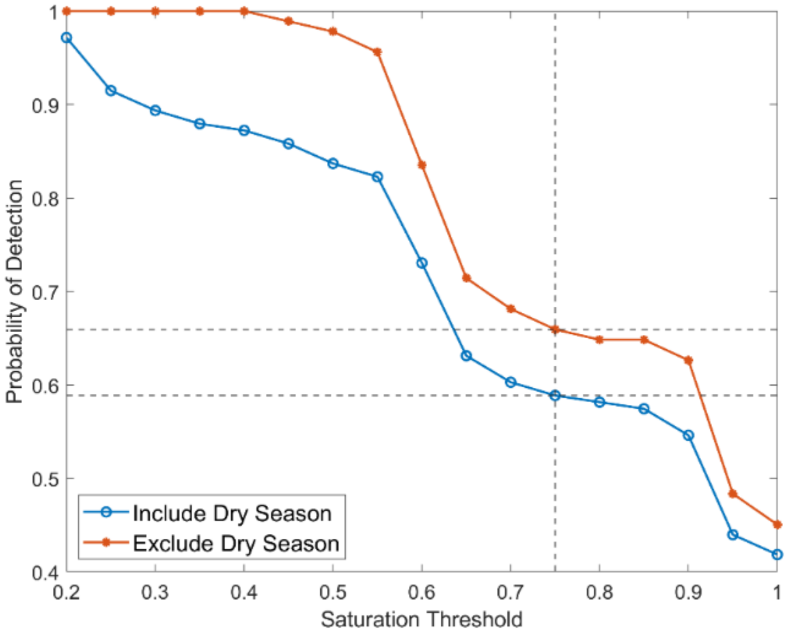

Figure S8. Sensitivity analysis of the probability of detection to saturation threshold. The probability of detection determines if the model can predict an aquatic habitat successfully and can be calculated based on the ratio of successful predictions to the total number of observations. The dotted vertical line corresponds to the selected threshold of 0.75, which results in a reasonable POD of 0.66 excluding dry season and a POD of 0.59 including dry season.

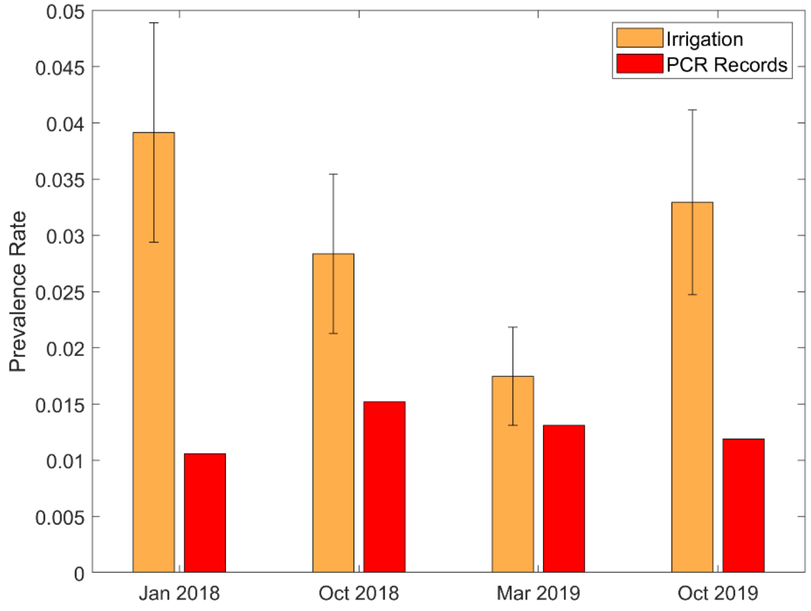

Figure S9. Comparison of simulated monthly average prevalence rate in Irrigation and measured prevalence diagnosed by Polymerase Chain Reaction (PCR). The whisker on the bar plot represents one standard error.

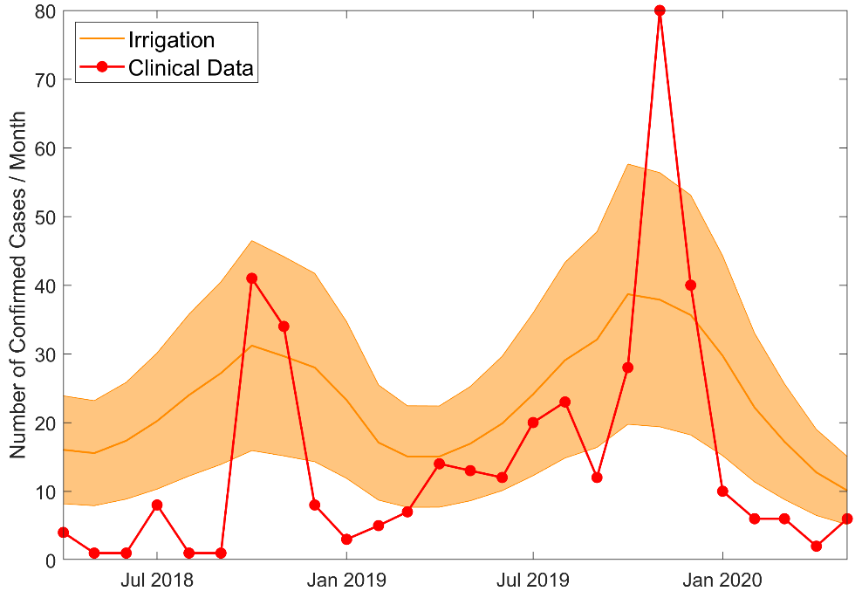

Figure S10. Comparison of simulated monthly confirmed cases in Irrigation and clinical data. The orange band indicates the 95% confidence interval.

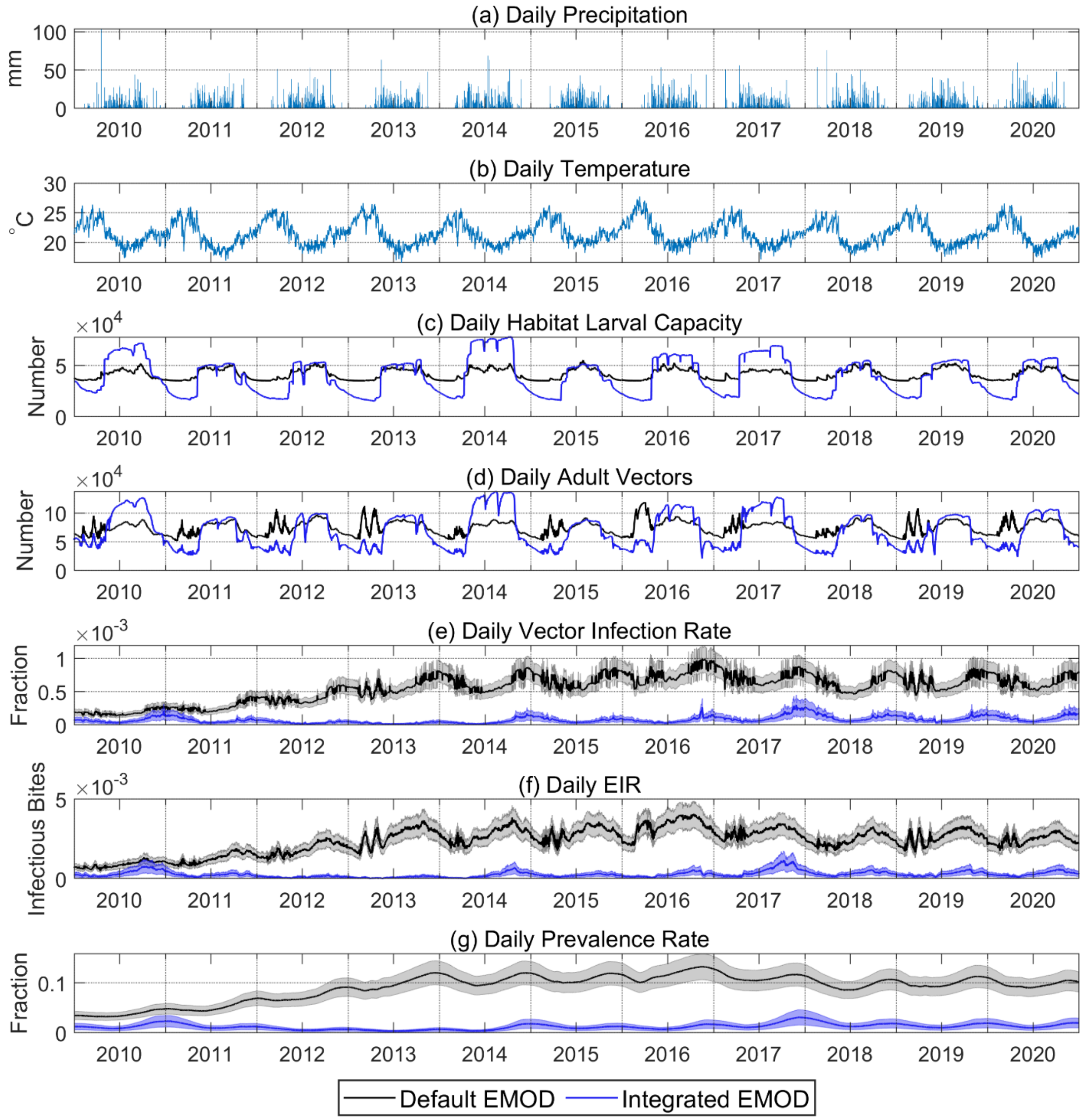

Figure S11. Time series of daily climate data and comparison of simulated daily malaria transmission results between Default EMOD and Integrated EMOD. We only show the truncated time series from 2010 to 2020 instead of the full simulation period for brevity and readability. Climate data include (a) precipitation and (b) temperature. Malaria transmission results include (c) habitat larval capacity, (d) adult vector abundance, (e) adult vector infection rate, (f) entomological inoculation rate and (g) parasite prevalence rate.

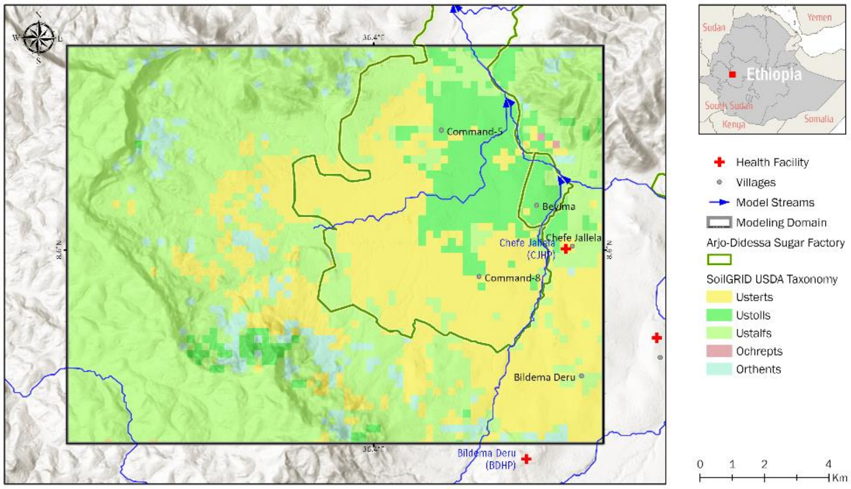

Figure S12. The distribution of the top two-meter soil types in USDA soil taxonomy from SoilGrids250m TAXOUSDA dataset. Most soil types in this area are characterized as clay or clay loam with low permeability ranging from 0.0015 to 0.015 m/h.

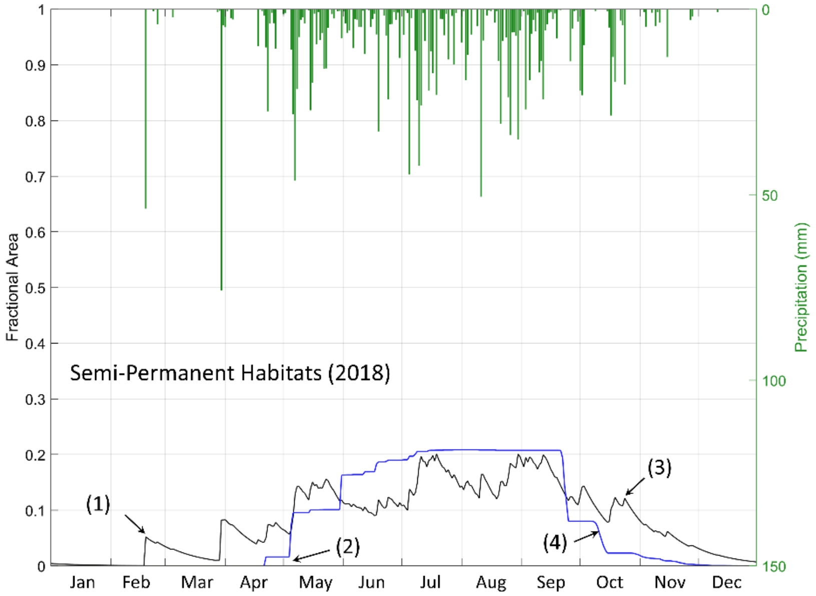

Figure S13. Comparison of simulated semi-permanent habitats between Default EMOD (black line) and Integrated EMOD (blue line) in 2018. Earlier rising limb in Default EMOD: (1) no infiltration, new ponds created instantaneously by rainfall; (2) ponds formed sometime after rainfall when soil saturation exceeded the threshold. Delayed falling limb in Default EMOD: (3) habitat area continued to increase with rainfall; (4) pond drained/dried up and soil became unsaturated after a period without rainfall, new rainfall insufficient to create ponding as soil saturation remained below threshold.

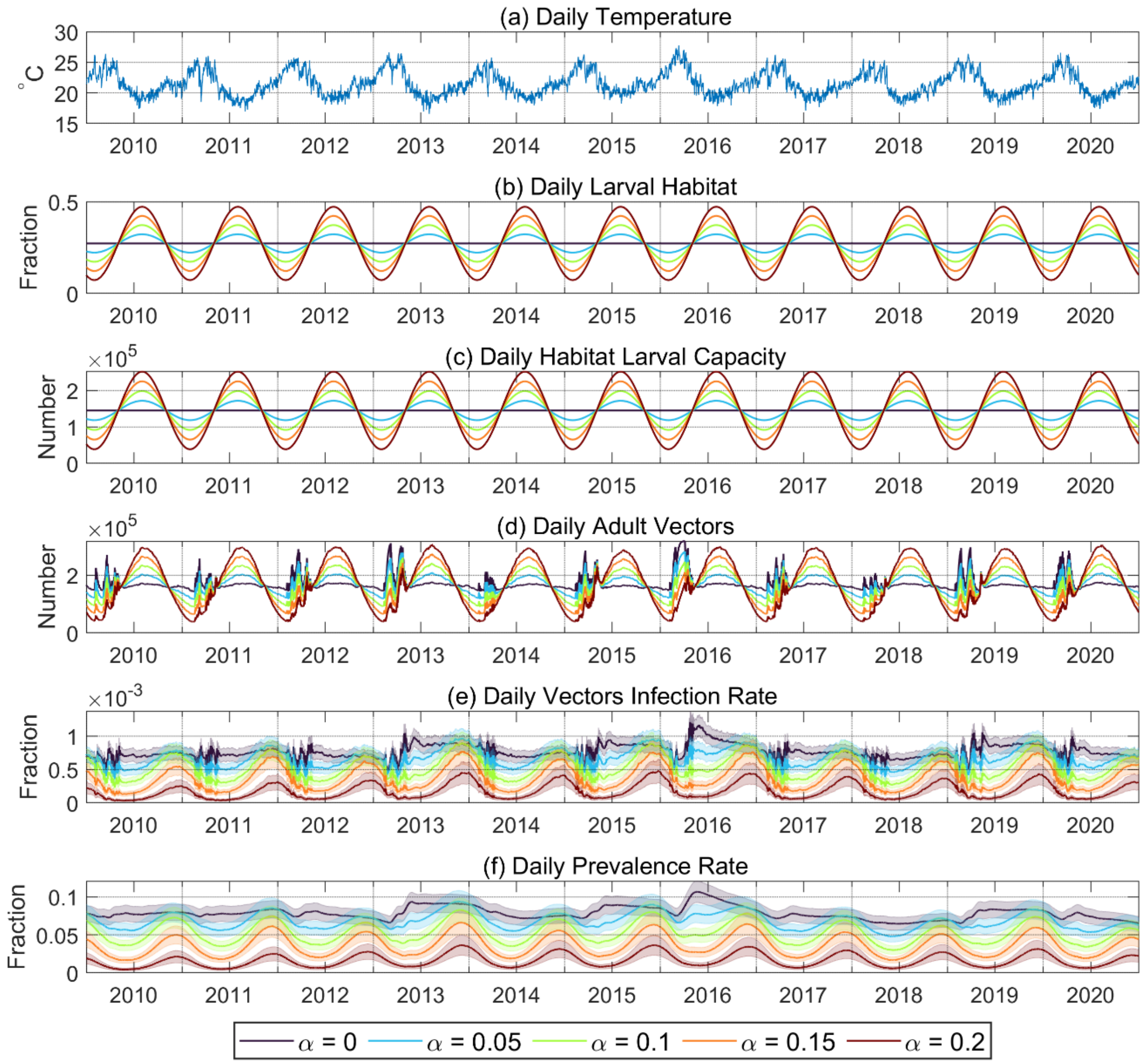

Figure S14. Simulation results from sensitivity analysis of malaria transmission to different amplitudes of larval habitat seasonality, α. Time series include (a) daily temperature, (b) synthetic sinusoidal larval habitat, (c) habitat larval capacity, (d) adult vector abundance, (e) adult vector infection rate and (f) parasite prevalence rate.

Table S1. Input data for ParFlow-CLM and EMOD.

Variable	Resolution	Latency	Source
**Topography**	5-meter	-	ALOS WORLD 3D Topographic Data (Takaku et al., 2016; Takaku & Tadono, 2017)
**Precipitation**	0.04°×0.04°, 3-hourly	~1 hour	Precipitation Estimation from Remotely Sensed Information using Artificial Neural Networks -Cloud Classification System- Climate Data Record (PERSIANN-CCS-CDR) (Sadeghi et al., 2021)
**Surface Solar Radiation Downwards**	0.25°×0.25°, 1-hourly	5 days	The Fifth Generation European Centre for Medium-Range Weather Forecasts Reanalysis (ERA5) (Hersbach et al., 2018, 2020)
**Surface Thermal Radiation Downwards**	0.25°×0.25°, 1-hourly	5 days	ERA5
**Air Temperature** (2m above ground surface)	0.25°×0.25°, 1-hourly	5 days	ERA5
**Skin Temperature**	0.25°×0.25°, 1-hourly	5 days	ERA5
**Surface Pressure**	0.25°×0.25°, 1-hourly	5 days	ERA5
**Water-vapor specific humidity**	0.25°×0.25°, 1-hourly	5 days	ERA5
**North-to-South Component of Wind Speed** (10m above ground surface)	0.25°×0.25°, 1-hourly	5 days	ERA5
**East-to-West Component of Wind Speed** (10m above ground surface)	0.25°×0.25°, 1-hourly	5 days	ERA5
**Land Use (2000)**	30-meter	-	Global Land Cover Mapping Project (GlobeLand30) (Chen et al., 2015)
**Land Use (2010)**	30-meter	-	GlobeLand30
**Land Use (2020)**	30-meter	-	GlobeLand30

**Soil Type**	250-meter	-	SoilGrids250m, TAXOUSDA (Hengl et al., 2017)
**Depth to Bedrock**	250-meter	-	SoilGrids250m, BDRICM (Hengl et al., 2017)
**Near Surface Permeability (< 100 m)**	Regional Scale	-	GLobal HYdrogeology MaPS 2.0 (GLHYMPS, 2.0) (Gleeson et al., 2014)

Table S2. Field data for ParFlow-CLM and EMOD validation.

Variable	Period	Number of Samples	Source
**Land Use**	July 2021	578	Site survey
**Larval Habitat**	2017-2021	769	Site survey
**Population**	2018-2021	-	Site survey
**Prevalence Rate**	January, October 2018; March, October 2019	4	Site survey
**Clinical Case**	April 2018-May 2020	26	Site survey

Table S3. Calibrated parameters in EMOD.

Parameter	Value
Antibody Memory Level	0.298
Base Sporozoite Survival Fraction	0.1667
Cytokine Gametocyte Inactivation	0.01335
Falciparum PfEMP1 Variants	150
Mean Sporozoites Per Bite	6
Merozoites Per Hepatocyte	990
Min Adapted Response	0.0174
Pyrogenic Threshold	500
Adult Life Expectancy	20
Male Life Expectancy	14

Aquatic Arrhenius 1	85,884,000,000
Aquatic Arrhenius 2	7,495
Infected Arrhenius 1	119,340,000,000
Infected Arrhenius 2	7,502
Scaling factor for larval capacity	1.66×10^{-5}

Table S4. Spatial average of adult vector from the dry season (November 2016 to April 2017) and the rainy season (May 2017 to October 2017).

**Scenario**	**Adult Vectors** (# /km^2)	
	**Dry Season**	**Rainy Season**
Default EMOD	697	800
Integrated EMOD (Non-Irrigation)	451	961
Irrigation	889	1,140

Table S5. Average simulated adult vector abundance, adult vector infection rate, and parasite prevalence rate for different amplitudes of larval habitat seasonality, α.

α	Adult Vectors	Vectors Infection Rate	Parasite Prevalence Rate
0.2	1.00	1.00	1.00
0.15	1.00	2.10	2.22
0.1	1.01	2.96	3.23
0.05	1.01	3.76	4.18
0	1.01	4.29	4.80

References

Bershteyn, A., Gerardin, J., Bridenbecker, D., Lorton, C. W., Bloedow, J., Baker, R. S., et al. (2018). Implementation and applications of EMOD, an individual-based multi-disease modeling platform. *Pathogens and Disease*, *76*(5), 1–10. https://doi.org/10.1093/femspd/fty059

Bill & Melinda Gates Foundation. (2023). Overview of EMOD software. Retrieved May 11, 2023, from https://docs.idmod.org/projects/emod-malaria/en/2.20_a/software-overview.html

Bill & Melinda Gates Foundation. (2023a). Larval habitat. Retrieved September 1, 2023, from https://docs.idmod.org/projects/emod-malaria/en/latest/vector-model-larval-habitat.html

Bill & Melinda Gates Foundation. (2023b). Vector transmission model. Retrieved September 1, 2023, from https://docs.idmod.org/projects/emod-malaria/en/latest/vector-model-transmission.html#vector-transmission-model

Bill & Melinda Gates Foundation. (2023c). Malaria symptoms and diagnostics. Retrieved September 1, 2023, from https://docs.idmod.org/projects/emod-malaria/en/latest/malaria-model-symptoms-diagnosis.html#fever-and-clinical-cases

Chen, J., Chen, J., Liao, A., Cao, X., Chen, L., Chen, X., et al. (2015). Global land cover mapping at 30 m resolution: A POK-based operational approach. *ISPRS Journal of Photogrammetry and Remote Sensing*, *103*, 7–27. https://doi.org/10.1016/j.isprsjprs.2014.09.002

Cornell University. (2010). PO 12.1-3 Field Capacity, Permanent Wilting Point & Available Water Capacity. Retrieved May 2, 2023, from https://nrcca.cals.cornell.edu/soil/CA2/CA0212.1-3.php

Eckhoff, P. A. (2011). A malaria transmission-directed model of mosquito life cycle and ecology. *Malaria Journal*, *10*(1), 1–17. https://doi.org/10.1186/1475-2875-10-303

Eckhoff, P. A., & Wenger, E. A. (2016). The EMOD Individual-Based Model. In S. M. Niaz Arifin, G. R. Madey, & F. H. Collins (Eds.), *Spatial Agent-Based Simulation Modeling in Public Health: Design, Implementation, and Applications for Malaria Epidemiology* (1st ed., pp. 185–208). John Wiley and Sons. https://doi.org/10.1002/9781118964385.ch11

Gleeson, T., Moosdorf, N., Hartmann, J., & van Beek, L. P. H. (2014). A glimpse beneath earth's surface: GLobal HYdrogeology MaPS (GLHYMPS) of permeability and porosity. *Geophysical Research Letters*, *41*(11), 3891–3898. https://doi.org/10.1002/2014GL059856

Hengl, T., Mendes de Jesus, J., Heuvelink, G. B. M., Ruiperez Gonzalez, M., Kilibarda, M., Blagotić, A., et al. (2017). SoilGrids250m: Global gridded soil information based on machine learning. *PLOS ONE*, *12*(2), e0169748. https://doi.org/10.1371/journal.pone.0169748

Hersbach, H., Bell, B., Berrisford, P., Biavati, G., Horányi, A., Muñoz Sabater, J., Nicolas, J., et al. (2018). ERA5 hourly data on single levels from 1959 to present.

https://doi.org/10.24381/cds.adbb2d47

Hersbach, H., Bell, B., Berrisford, P., Hirahara, S., Horányi, A., Muñoz-Sabater, J., et al. (2020). The ERA5 global reanalysis. *Quarterly Journal of the Royal Meteorological Society*, *146*(730), 1999–2049. https://doi.org/10.1002/qj.3803

Hinne, I. A., Attah, S. K., Mensah, B. A., Forson, A. O., & Afrane, Y. A. (2021). Larval habitat diversity and Anopheles mosquito species distribution in different ecological zones in Ghana. *Parasites and Vectors*, *14*(1), 1–14. https://doi.org/10.1186/s13071-021-04701-w

Kollet, S. J., & Maxwell, R. M. (2006). Integrated surface–groundwater flow modeling: A free-surface overland flow boundary condition in a parallel groundwater flow model. *Advances in Water Resources*, *29*(7), 945–958. https://doi.org/10.1016/J.ADVWATRES.2005.08.006

Kuffour, B. N. O., Engdahl, N. B., Woodward, C. S., Condon, L. E., Kollet, S., & Maxwell, R. M. (2020). Simulating Coupled Surface-Subsurface Flows with ParFlow v3.5.0: Capabilities, 2 applications, and ongoing development of an open-source, massively parallel, integrated 3 hydrologic model. *Geoscientific Model Development*, *13*(3), 1373–1397. https://doi.org/10.5194/gmd-2019-190

Kweka, E. J., Zhou, G., Munga, S., Lee, M. C., Atieli, H. E., Nyindo, M., et al. (2012). Anopheline Larval Habitats Seasonality and Species Distribution: A Prerequisite for Effective Targeted Larval Habitats Control Programmes. *PLoS ONE*, *7*(12), e52084. https://doi.org/10.1371/journal.pone.0052084

Maxwell, R. M., & Miller, N. L. (2005). Development of a Coupled Land Surface and Groundwater Model. *Journal of Hydrometeorology*, *6*(3), 233–247. https://doi.org/10.1175/JHM422.1

Sadeghi, M., Nguyen, P., Naeini, M. R., Hsu, K., Braithwaite, D., & Sorooshian, S. (2021). PERSIANN-CCS-CDR, a 3-hourly 0.04° global precipitation climate data record for heavy precipitation studies. *Scientific Data*, *8*(1), 1–11. https://doi.org/10.1038/s41597-021-00940-9

Takaku, J., & Tadono, T. (2017). Quality updates of 'AW3D' global DSM generated from ALOS PRISM. In *2017 IEEE International Geoscience and Remote Sensing Symposium (IGARSS)* (pp. 5666–5669). IEEE. https://doi.org/10.1109/IGARSS.2017.8128293

Takaku, J., Tadono, T., Tsutsui, K., & Ichikawa, M. (2016). Validation of 'AW3D' Global DSM Generated from ALOS PRISM. *ISPRS Annals of the Photogrammetry, Remote Sensing and Spatial Information Sciences*, *III–4*, 25–31. https://doi.org/10.5194/isprsannals-III-4-25-2016
